# Clinical Importance of miRNA in Diabetic Neuropathy: Pathophysiology, Diagnosis, and Therapeutic Potential

**DOI:** 10.2174/0115733998375284250905115146

**Published:** 2025-09-23

**Authors:** Desh Deepak Singh

**Affiliations:** 1 Amity Institute of Biotechnology, Amity University Rajasthan, Jaipur, 303002, India

**Keywords:** Diabetic neuropathy, microRNA, pathophysiology, neurovascular dysfunction, therapeutic potential, microvascular endothelial communication networks, neurodegenerative disease

## Abstract

Diabetic Neuropathy (DN) is the major chronic complication in diabetic patients. The exact pathophysiological mechanisms of DN are not fully understood; however, failures in axon–Schwann cell and microvascular endothelial communication networks play major roles in DN progression. The multiple pathophysiological mechanisms of DN are regulated by microRNAs (miRNAs), including inflammation, vascularization, angiogenesis, posttranscriptional regulation, intercellular communication, and signalling pathways. Various types of miRNA affect the gene expressions within cells, but their profiles often change during DN, including SMAD, PI3K, Nuclear Factor kappa B (NF-κB), and MAPK. DN has been associated with the miRNAs-9, miRNA-106, miRNA-182, miRNA-23a, miRNA-23b, miRNA-23c, miRNA-503, miRNA-203, miRNA-145, and miRNA-126. MiRNA dysregulation is one of the first molecular changes seen in diabetics. Therefore, miRNAs hold promise as both therapeutic targets and diagnostic biomarkers. This study aims to discuss the importance of miRNA in clinical pathophysiology, diagnosis, signalling pathways, and therapeutic targets for DN.

## 1 INTRODUCTION

The most prevalent long-term consequence of diabetes mellitus is DN. The pathophysiological mechanism of DN is still challenging in diabetic patients [1]. MiRNA (micro RNA) regulates the cell signalling mechanism and has been involved in the pathophysiological condition of DN by modifying the cellular and biochemical processes and impacting the severity of diseases [2]. MiRNA is a single-stranded RNA with 19-24 nucleotide base sequences that regulates the expression of genes within the cell (Fig. **1**) [3]. MiRNAs are processed and matured by the microprocessor complex, which is composed of nuclear RNase III Drosha and cofactor DGCR8 (DiGeorge syndrome critical region 8) [4]. RNase III Drosha cleaves precursor miRNAs into ~65 base pair hairpin structures, which are then transported to the cytoplasm. There, Dicer endonucleases process them into ~21 nucleotide fragments that associate with GW182 proteins to form the miRNA-induced silencing complex (miRISC) (Fig. **2**) [4]. The miRISC proteins work by recruiting mediators that enhance mRNA deadenylation and inhibit miRNA translation. miRNAs regulate diverse cellular processes, including cell growth, differentiation, immune responses, hyperglycemia, and vascularization [5]. The clinical importance of molecular pathways in the development of DN varies with the distinct cell type, disease profile, and time of the metabolic impairments [6]. Furthermore, some other pathways are also involved in the pathogenesis of DN, including PKC (protein kinase C), hexosamine, and polyol. Glycolysis in the polyol route produces fructose and sorbitol, which inhibit Na^+^ and K^+^ ATPase action due to elevated sorbitol levels [7]. Glucose is changed to fructose-6-phosphate, and glyceraldehyde-3-phosphate enters the hexamine pathways, AGEs (Advanced Glycation End Products), and PKC (Protein Kinase C) pathways by the activation of PARP signalling [7]. Due to impairment in TCA and oxidative phosphorylation, this leads to overload of the electron transport chain, resulting in mitochondrial dysfunction [8]. ROS is generated by osmotic stress, which leads to DNA damage and endoplasmic reticulum stress [9]. MiRNA can induce mRNA degradation, alter the expression profile, block the translation process, and lead to the development of clinical conditions of DN [10]. Recently, diabetic patients’ miRNA profiles have been reported; miRNA have been investigated in 63 DN patients of mononuclear cells [11]. Peripheral nuclear cells have shown expression of miRNA-125P, miR-145-3p, and miRNA-191-5p (Fig. **2**). DN was more likely to develop in patients with the miRNA-499a rs374644 GG genotype [12]. The miRNA network is established by analyzing miRNA-346, miRNA-330P, and miRNA-17-1-3p expression profiling in the DN. MiRNA-181a-1, miRNA-541, miRNA-341, and miRNA-203 are deregulated in an animal model of DN [13]. The miRNAs are involved in DN and can be used as diagnosis and therapeutic targets [14]. The correct regulations and main function of the signalling molecules at the transcriptional and translational levels still need to be explored. Reducing the clinical complications during disease progression and prevention remains the major diagnostic and therapeutic approach in DN [15]. Therefore, clinical measures can only be achieved by the exploration of pathophysiology in DN *via* prevention of neuropathy acceleration and/or activation of neuropathy-suppressing signalling mechanisms. Data searches were conducted using Google, PubMed, Scopus, Google Scholar, and Web of Science. This study aims to discuss the role of miRNAs in the clinical pathophysiology, diagnostics, signaling pathways, and therapeutic targets of DN.

### 1.1 MiRNA in Inflammation of DN

DN is an exclusive neurodegenerative disease, and it mainly damages the sensory axons, peripheral axons associated with Schwann cells, and, later on, damages motor neurons [16]. Damage to these cells has demonstrated a range of alterations, including mRNA, spliceosome functions, changes in the expression of motor neurons, reduced blood flow, and miRNA alterations in DN [2]. DN is a complex condition that arises from chronic high blood sugar levels, impacting both the peripheral and autonomic nervous systems [17]. It is marked by the gradual damage of nerve fibres, which results in sensory, motor, and autonomic dysfunction [18]. The underlying mechanisms of DN include metabolic, vascular, inflammatory, and neurodegenerative factors that contribute to nerve injury [8]. Chronic hyperglycemia activates various biochemical pathways that lead to oxidative stress, mitochondrial dysfunction, and impaired nerve function [19]. Insulin regulations are altered by hyperglycemia, and dyslipidaemia leads to pathological modifications in vascular cells, glia, and neurons, which can lead to dysfunctions of these cells and loss of neurotropic signalling and the development of DN [20]. Aldose reductase and Sorbitol Dehydrogenase (SD) play a major role in the metabolism of glucose *via* the polyol pathway; by using SD and NAD+ (nicotinamide adenine dinucleotide), they oxidize sorbitol to fructose [21]. AR demonstrates lower affinity at low percentages of glucose, which is metabolized by the polyol pathway; during hyperglycaemic situations, more glucose is converted to sorbitol *via* the enzymatic reduction process, with a reduced NADPH (Nicotinamide adenine dinucleotide phosphate) process [22]. The polyol pathway begins with aldose reductase converting glucose into sorbitol, which is subsequently oxidized by sorbitol dehydrogenase into fructose. The buildup of sorbitol and fructose creates osmotic stress, causing swelling in axons and Schwann cells [23]. This process depletes NADPH, which reduces glutathione (a key antioxidant), thereby increasing oxidative stress. It also lowers myoinositol levels, negatively affecting nerve conduction [24]. AGEs are formed as a result of chronic hyperglycemia, leading to the non-enzymatic glycation of proteins and lipids. AGEs bind to their receptors (RAGE), which triggers inflammation through the activation of NF-κB, TNF-α, and IL-6. Additionally, oxidative stress (marked by increased RIS production) and endothelial dysfunction reduce blood flow to the nerves [25]. Mitochondrial dysfunction and oxidative stress are significant contributors, as mitochondria are an essential energy source for neurons [26]. High blood sugar levels disrupt the Electron Transport Chain (ETC), leading to an increase in Reactive Oxygen Species (ROS). This ROS production causes lipid peroxidation, damaging myelin and axons [27]. Furthermore, DNA damage activates pro-apoptotic pathways and disrupts protein function, impairing cellular repair and energy metabolism [28]. PKC becomes overactive due to elevated Diacylglycerol (DAG) levels in hyperglycemia. PKC plays a role in reducing nitric acid production, which leads to vasoconstriction and nerve ischemia [29]. This results in increased vascular permeability, causing nerve oedema, neuroinflammation, and impairing neuronal function. Vascular dysfunction, characterized by ischemia and hypoxia, develops as peripheral nerves rely on small blood vessels for oxygen and nutrients [17]. Changes in the vasculature due to hyperglycemia include endothelial dysfunction, where decreased nitric oxide results in vasoconstriction. A thickened capillary basement membrane reduces oxygen diffusion and increases vascular permeability, contributing to nerve oedema and compression [17]. Hypoxia also develops, leading to axonal degeneration and Schwann cell dysfunction. Hypoxia-Inducible Factor (HIF-1α) typically protects nerves from hypoxia, but in diabetes, its downregulation means nerves are more susceptible to ischemic injury [30]. Chronic low- grade inflammation induced by hyperglycemia plays a significant role in nerve injur, with the NLRP3 inflammasome being activated [31]. In diabetes, the NLRP3 inflammasome, crucial immune sensors, is over activated, resulting in increased levels of IL-1β and IL-18, which amplify inflammation and enhance the activation of macrophages and microglia, further exacerbating nerve damage [32]. There are also alterations in ion channel function and pain sensitization, as neurons depend on ion channels to transmit signals. In DN, the function of these channels is impaired, with deregulation of sodium (Na^+^) and calcium (Ca^2+^) channels. Increased Na^+^ channel activity leads to spontaneous nerve firing and neuropathic pain, while elevated intracellular Ca^2+^ contributes to mitochondrial stress and apoptosis [33]. Diabetic-related transgenic mice with increased AR expression in Schwann cells demonstrated a higher MNCV (motor nerve conduction velocity) decrease in the peripheral nerves [34]. Schwann cells are damaged by osmotic pressure due to high intracellular sarbital concentration [35]. The accumulation of fructose and sorbital leads to a reduction in the level of taurin, myoinositol, and glutathione, which inhibits the sodium potassium adenosine triphoapahtase pump, which may lead to the accumulation of sodium ions, inflammations in the axon, and reduced nerve conduction activity [8]. AR (aldose reductase) activity is increased in hyperglycemia and oxidative-nitrosative stress, which leads to upregulation of protein kinase and the cell signalling pathways PARP and NF-κB [36]. Activity in dorsal root ganglia, spinal cord, and nerve cells is regulated by Mitogen-Activated Protein Kinase (MAPK) pathways in diabetic patients [37]. However, there is a need to explore key miRNAs and target them as pathophysiological conditions of DN in diabetic patients (Table **1**) [14]. Novel findings on the clinical importance of miRNAs in Diabetic Neuropathy (DN) pathophysiology based on recent studies (Table **2**) [2].

### 1.2 MiRNA in Inflammation of DN

DN is linked with innate immune system activation and inflammation; during hypoglycemic conditions, it increases the fibronectin, ICAM-1 (intracellular cell adhesion molecules-1), transforming Growth Factor Beta (TGF-β), Interleukin-6 (IL-6), Vascular Endothelial Growth Factor (VEGF), NF-κB, and Tumour Necrosis Factor Alpha (TNF-α) [50]. MiRNAs are involved in neuropathy inflammation, TNF-α from neuropathic tissue, NF-κB, Monocyte Chemoattractant Protein-1 (MCP-1), macrophage M1, and the release of cytokines [51]. Furthermore, miRNA-21 and miRNA-214 target the PTEN (phosphatase and tensin homolog), which regulates the NF-κB signalling and controls the inflammatory gene expression during DN [5]. Vascular complications occur due to damage to the endothelial cells in hyperglycemia. These regulations may cause deregulation of the nervous system. Researchers have investigated miRNA-mediated neuropathy [52]. MiRNA-9 stimulated the ATP-P2X7R (adenosine 5’-triphosphate (ATP)-triggered signalling through the ionotropic purinergic receptor 7) signalling mechanism in the spinal cord of rats by interacting with calcium homeostasis modular-1 (CALHM1) [53]. Neuropathic pain is regulated by miRNA-155 after NF-κB activation inhibition, which reduces the level of inflammatory cytokines [54]. Members of the solute carriers family SL17A6 reduce the expression level of IL-1b and IL-6 in the lumbar spinal dorsal horn [55]. MiRNA-146 expression is also upregulated in DN [55]. The treatment with miRNA-146a mimics improved nerve conduction and the footpad of DN mice [46]. MiRNA-146 changes the polarization of macrophages by elevating the activation of anti-inflammatory macrophages and lowering the activation of pro-inflammatory macrophages [46].

### 1.3 MiRNA in Neurovascular Dysfunction

The DRG neuron is damaged, and changes in mRNA and miRNA expression occur due to oxidative stress, inflammation, AGE buildup, and mitochondrial dysfunction [17]. In a mouse model for DN, structural abnormalities and neurological dysfunction are separately elevated by the let-7i mimic and anti-miRNA-341 [56]. MiR-29b is discovered to be downregulated in DRG neurons of STZ-induced diabetic mice [57]. In DRG, axonal swelling, apoptosis, and aberrant gene expression profiles are linked to reduced miR-29b [57]. MiRNA-29C is upregulated in the peripheral nervous tissue of diabetic mice [58]. A significant reduction in axonal development has been shown in DRG neurons grown under the condition of hyperglycemia [58]. However, reduction of endogenous miRNA-29c boosted axonal development when the DRG neurons were transected with inhibitors of miRNA-29C (Fig. **3**) [58]. Axonal miRNA controls the local regulation of energy metabolism [59]. High glucose locally reduced the level of miRNA-146 in the distal axon of DRG neurons cultured in a microfluidic device [60]. Through its target genes, miRNA-146a regulates axonal growth through gain and loss of function (Fig. **3**).

### 1.4 MiRNA in Schwann Cells

MiRNAs have been shown to be involved in the Schwann cell-axon interaction and the Schwann cell response to injury using *in vitro* and *in vivo* DN models [61]. Dicer, a vital enzyme required for miRNA development, has been associated with Schwann cell myelination, according to a recent investigation [62]. Notch 1 and cyclin D-mediated differentiation could potentially be controlled by miR-34a [63]. It has been investigated that miR-140 influences the production of Krox 20, a transcriptional factor associated with myelin genesis [64]. In addition, by targeting specific neural factors, Let-7d markedly decreased basic Schwann cell proliferation and migrations [2]. MiRNA-9, miR-sc3, and miRNA-30c have been involved in the migration and proliferation of Schwann cells [2]. Furthermore, by treating specific nerve growth factors, Let-7d substantially reduced the proliferation of primary Schwann cells [61]. Circular RNA-induced reduction of miR-145-3p in cultured rat Schwann cells (RSC96) reduced ROS (reactive oxygen species) production [65]. Autophagy and high glucose-induced cell death are regulated by the PI3K/AKT/mTOR pathway [66]. Consequently, it may be possible to employ Schwann cell miRNA modifications therapeutically to counteract the consequences of DN.

### 1.5 Microvascular Dysfunction and miRNA

Diabetes is believed to arise from a microvascular complication with DN [67]. There are various ways in which hyperglycemia induces cellular damage in the vascular endothelium. Patients with diabetic neuropathy exhibit thicker capillary basement membranes and capillary pericyte degeneration in their nerve biopsy [68]. Microvasculature dysfunction either precedes or coexists with nerve dysfunction and demyelination, reducing nourishment, oxygen, and waste delivery across the nervous system [69]. In comparison to control volunteers, miR-199a-3p was considerably greater in research involving sixty patients with DN [41]. The miR-199a-3p increases skin peripheral circulation and coagulation by up-regulating endothelial cell MMP-13 and down-regulating serine protease inhibitor E2 (Serine E2) [41]. These investments highlight the role of miRNAs in DN control by increasing blood flow, which in turn improves neuronal function.

### 1.6 MiRNA in Neuropathic Pain

The first evidence report on the relationship between pain and microRNAs was published in 2007, which identified seven dysregulated miRNAs following CFA (Complete Freund's Adjuvant) injection in the master muscles of the rats [70]. The nociceptor-specific deletion of Dicer, an enzyme responsible for pre-miRNA cleavage, resulted in a lower response to CFA intraplanar injection compared to wild-type [71]. Dicer null mice exhibited altered mRNA transcripts, including pain-related markers. Surprisingly, these transcripts are downregulated, indicating that the loss of miRNA may lead to the upregulation of transcriptional repressors, which control the expression of these nociceptive genes [72]. Various types of miRNA and their targets have been identified in animal models of neuropathic pain [73]. Computational models are anticipated miRNA targets, but experimental validation is required to authenticate their pathophysiological importance [74]. Validation of target grips mRNA/miRNA link, co-expression *via* RT-PCR on *in situ* hybridization, and impact on target protein function through miRNA overexpression or knockdown [75]. The identified miRNAs and their potential targets are involved in a wide range of pain-associated pathways, which can be roughly characterized as neuronal excitability, intracellular signaling, and contact with non-neuronal cells [17]. The investigation of miRNA in pain has helped us understand how miRNA levels are modulated.

### 1.7 Diagnostic Importance of miRNA in Diabetic Neuropathy

Qualitative and quantitative methods are used in the diagnosis of DN. Patients mainly undergo imaging tests, such as CT (computed tomography) scans and MRI (magnetic resonance imaging), as well as blood testing for diabetes, vitamins, and immunological biomarker investigations [76]. Furthermore, some aspects of investing are also evaluated, including weak reflexes, numbness, and muscle weakness. There are various diagnostic biomarkers that have been investigated during the progression of DN in diabetic patients [77]. The expression of miR-190-5p, miR-126, miR-210, and miR-27 is upregulated, and the expression of miRNA-4448, miR-338-3p, miR-485-5p, miR-20b, and miR-17-3p is downregulated in the serum samples of DN patients [78]. MiRNA-93 has been observed to be upregulated in the plasma samples, and miRNA-29b is downregulated in the plasma samples of DN patients [79]. MiRNA-221 is upregulated in the peripheral blood endothelial progenitor cell cultured *in vitro* [80]. The miR-203a and miR-200b-3p have shown downregulation in the retinal vascular endothelial cells of DN patients [81]. The miRNA-106a-5p and miRNA-15a are observed as upregulated, while miR-222, miR-20a-5p, and miR-20a-5p are downregulated in the circulatory extracellular vesicles of DN patients [82]. MiR-204 is upregulated, while miRNA 199a-5p, miR-204, miR-16, and miR-92 have been observed downregulated in the vitreous humour of DN patients [83]. These findings suggest that miRNA could be a useful biomarker for DN. Novel findings on the clinical importance of miRNAs in DN diagnosis based on recent studies are shown in Table **3**.

### 1.8 MiRNAs and Extracellular Vesicles

Since miRNA has been found in biological fluids, investigators have examined the features of these molecules in order to evaluate whether they may be used as biomarkers or as targets for treatment [92]. MiRNA can withstand RNase degradation through two potential mechanisms: forming a complex with argonaute protein or being encapsulated in lipoproteins or Extracellular Vesicles (EVs) [93]. Exosomes exhibit high nucleic acid loading capacity, low toxicity, and low immunogenicity. They therefore have considerable potential as therapeutic vehicles. It has been discovered that exosomal miRNAs control target gene expression, which in turn mediates physiological responses in the receiving cells [94]. Employing dicer silencing, some research produced EVs depleted of miRNA and eliminated the impact of naïve exosomes [95]. These findings imply that the majority of the molecular and biological impacts of exosomes are mediated by their mRNA cargo. Exosomal mRNAs are being used in a number of active clinical trials to diagnose or investigate the pathogenesis of myocardial infarction, diabetic retinopathy, cancer, and sepsis [96].

### 1.9 Axonal Growth Regulatory miRNAs

DRG axons have been internalized by exosomes, which are made by Schwann cells to improve axonal regeneration in a preclinical study [97]. Axonal and myelin degeneration is reduced by the use of exosomes derived from Schwann cells, which in turn reduced neuropathic symptoms in DN mice [98]. Conversely, by reducing epidermal nerve fibers, exosomes derived from Schwann cells cultured in a high-glucose medium accelerated the development of diabetic neuropathic pain [99]. MiR-21, miR-27a, and miR-146a are expressed in naïve Schwann cells. The loss of miR-27 in exosomes removes their ability to promote the migration of Schwann cells and axonal growth of diabetic DRG neurons [100]. It has been discovered that the genes miR-21, miR-27a, and miR-146a target genes, such as NF-κB, PTEN, RhoA (Ras homolog family member A), SEMA6A (Semaphorin-6A), and the Ras homolog gene family [2]. High glucose-stimulated Schwann cell exosomes have been shown to be enriched in miR-28, miR-31a, and miR-130 [99]. These miRNA regulations specifically target proteins involved in axonal growth, such as growth-associated protein 25 and methyltransferase-3a.

### 1.10 Regulations of miRNA Expression and Their Therapeutic Potential in DN

DN is a multifaceted condition with intricate pathogenic molecular mechanisms that remain to be understood [101]. DN is improved by applying AR inhibitors, including spirohydantoins and associated cyclic amides (fidarestat, soabinil) and carboxylic acid derivatives (zopolrestat, zenarestat, torestat, ponalrestat, lidorestat, epalrestat, and alrestatin), with the reduction of sorbitol production, enhancing the blood flow in the nerve, and restoring MNCV [102]. The underlying molecular pathways discussed here include metabolic failure, inflammation, oxidative stress, TGF-β signalling, the PI3K/Akt signalling pathway, and the ubiquitin-proteasome during DN [103]. Clinical complications of DN also control its miRNA regulations, and therefore, it may be a potential target for treatment [104]. This section discusses the roles of different molecular pathways and their clinical importance in DN.

### 1.11 MiRNA Regulations in the TGF-β Signalling Pathway

TGF-β is an important pathway in DN and plays an important role in ECM (extracellular matrix accumulation) and glomerular hypertrophy [104]. TGF-β is involved in AKT kinase (protein kinase B) through inhibition of PTEN, MAPK (mitogen-activated protein kinases, and ERKS (Extracellular signal-regulated kinases) in diabetes [32]. These signalling pathways and those linked between them can amplify and induce the expression of DN-related pathological genes. Recent investigations have shown that various miRNAs regulate the TGF-β signalling pathways and can be clinically important in DN pathogenesis [105]. SMAD3 auto-regulations increase the TGF-β1 expression through miRNA-192. ZEB2 (E box binding homeobox 2) is also inhibited by miR-192, which is mediated by the expression of COL1α1 and COL1α2 [106]. In addition, ZEB-1 gene expression is inhibited by attachment to the 3’UTR of its miRNA-200b and miR-200C. MiR-200 and MiR-216a target Y-box binding protein 1 (Ybx1) or Y-box transcription factor 1 directly or simultaneously function as a promoter of DN to unregulated TGF-β1 expression in renal MCs [107]. MiR-21 is upregulated in diabetic patients with DN. miR-195 reduces DN through an array of approaches by inhibiting sirtuin 1 (Sirt 1), a NAD-dependent deacetylase enzyme in DN, through various methods [14]. Various miRNAs have been described as negative regulators of the TGF-β1 signalling pathway in DN, including miR-30c, miR-200a, and miR-141 [108]. However, by understanding the clinical importance of the above miRNAs in DN, therapeutic options can be developed.

### 1.12 MiRNA in Fibronectin and Collagen Gene Expression in DN.

Collagen and Fibronectin (FN), two important characteristics of DN, are crucial for the thickening of the glomerular membrane and the expansion of the mesangial matrix as a result of an accumulation of these ECM proteins [109]. It has been observed that the expression of the collagen gene in DN is influenced by two families’ miRNA [14]. DN may be controlled more effectively through modifying the level of expression of collagen by a novel therapeutic potential that blocks DN-inducing miRNAs and increases DN-inhibiting miRNAs [104].

### 1.13 MiRNA in the PI3K/Akt Signalling Pathway in DN

MiRNA regulates the PI3K/Akt signalling pathway, which is linked to pathophysiological changes in DN, including mesangial cells, glomerular hypertrophy, and podocyte death [110]. MiRNA-217 and miRNA-216a activate the Akt signalling pathway by PTEN, which acts as a negative regulator for the PI3K/Akt signaling mechanism in the glomeruli of diabetic mice [42]. Akt regulation is mediated by PTEN dephosphorylating phosphatidylinositol 1,4-triphosphate; this mechanism is regulated by miRNA-21 in DN [111]. However, miRNA sponge, performing as a miRNA-21 inhibitor, reduces PTEN suppression in MCs, consequently attenuating PI3K/Akt signaling [110]. The overexpression of the miRNA-214 target PTEN causes an excessive activation of Akt signaling, which in turn leads to renal hypertrophy in both proximal tubular epithelial cells and renal glomerular meningeal cells [112]. MiRNA-200b/c is overexpressed in the DN mouse model, which is regulated by the PI3K/Akt signaling pathway [14]. FOG2 (Friend of GATA 2) inhibits PI3K/Akt activation by directly interacting with p85α, which is known to be a regulatory subunit of PI3K [113]. MiRNA upregulation stimulates Akt/FoxO3a (Forkhead box class O 3a) signalling and causes glomerular hypertrophy in the glomeruli of DN mice [114]. Given the significance of PI3K/Akt signalling in the pathophysiology of DN, DN may be attenuated by inhibiting the miRNA responsible for AKT activation.

### 1.14 The Ubiquitin-Proteasome System (UPS) and miRNAs in DN

The Ubiquitin-Proteasome System (UPS) plays a major role in disease development, including obesity, diabetes, and insulin resistance [115]. More than 80% of proteins are degraded by deactivating intracellular inhibitors [116]. Due to modifications in the UPS mechanism, it increases the activity of proteosome activator 28, 26S proteosomes, and immunoproteosomes, which may lead to DN [117]. Recent investigations have shown the direct involvement of miRNA in the UPS pathway in DN [14]. MiRNA-192 is upregulated in the E3 ubiquitin ligase expression in human proximal epithelial cells, which leads to renal fibrosis in DN progression by regulating UPS [118]. MiRNA-21 is highly expressed in DN and leads to tubulointerstitial fibrosis in mice by mediating the ubiquitination of SMAD7. MiRNA-23a initiates the ubiquitin degradation of SnoN and impairs the fibrogenesis in DN [119]. The Wnt/β-catenin signaling pathway activates the expression of UCHL1 (Ubiquitin C-terminal hydrolase L1), which is regulated by miRNA-193a [120]. Taking into account these investigations, the pathophysiological importance of miRNA in UPS activity suggests that proteosomes could be used as therapeutic opportunities in DN treatment.

### 1.15 MiRNAs and Molecules Linked to DN

Recent investigations have shown that the pathophysiology of DN may involve a few newly identified molecules [121]. MiRNA-regulated in DN that has nephrotoxic or nephroprotective potential is regulated by these molecules. Kidney tissue inhibition of metalloproteinase-3 (TIMP3) is highly expressed and has been identified as a regulator of fibrosis and inflammation [122]. It has been demonstrated that loss of TIMP-3 in diabetic mice enhances kidney oxidative stress, glomerular mesangium, and Metalloproteinase-2 (MMP-2) stimulation; all these factors are involved in the pathogenesis of DN [123]. In addition, by attaching to the 3’ UTR of Peroxisome Proliferators-Activated Receptor alpha (PARP-α), miRNA-21 downregulates the expression of PARP-α, which results in reduced fatty acid metabolism, increased expression of α smooth muscle actin, and kidney fibrosis. By attaching to the 3’ UTR of RECK mRNA, miRNA-21 targets endogenous MMP inhibitors and RECK (reversion-inducing cysteine-rich protein with Kazal motifs), activating MMPs and causing renal fibrosis [124]. Consequently, miRNA-21 may have various roles in the pathophysiology of DN; *via* Sirt-1 suppression, miRNA-217 can potentially modulate the expression of TIMP3. Overexpression of miRNA 181-5p, which targets the 3’ UTR of TNF-α mRNA, may attenuate inflammation and fibrosis in DN. It has been shown that in human HK2 cells exposed to miRNA-326 [85].

### 1.16 Approaches for Targeting miRNAs Therapeutically in DN

The emphasis is turning to the use of or inhibition of specific miRNAs, which are essential to the development of DN [14]. It is true that one possible therapy for DN is to upregulate kidney-protective miRNAs and downregulate miRNAs that induce DN [104]. Anti-MiRNA Oligonucleotides (AMOs), miRNA inhibitory agents, and miRNA sponges can all be used to block miRNAs, while miRNA inducers and miRNA analogues can be used to upregulate miRNAs. Novel findings on pathophysiological processes in DN and their interactions with specific molecular mechanisms are shown in Table **4**.

### 1.17 Mi-RNA Inhibitory Agent

It has been investigated that a few small molecules block miRNA. Resveratrol and curcumin are believed to be powerful down-regulators of miRNA-21 [85]. Small molecules, like glyceollins and garcinol, down-regulate miRNA-21 in pancreatic cells [131]. MiRNA-21 promotes DN through a variety of pathways [85]. In EC, miRNA-210 upregulates VEGF and VEGFR2 (Vascular endothelial growth factor receptor 2), while downregulating ephrin-A3, which promotes renal angiogenesis [132]. This suggests that Targapremir-210 could be a potential target in DN. These findings indicate that DN is promoting a novel treatment option [17]. There are the most common nucleotide modifications: locked nucleic acid, 2’-fluoro (2’ F) chemistries, 2’-o-methoxiethyl (2'MOE), and 2’-o-methylation (2’OMe). It has been shown that antagomir of miRNA-27a reduces renal fibrosis [133]. Antagomir of miRNA-132 has been shown to reduce tubular apoptosis by inhibiting the production of pericytes and myofibroblasts [134]. Antagonism of the miRNAs listed herein may have a potentially beneficial impact for the treatment of DN [14]. A group of bicyclic RNA analogues where the ribose ring is attached to a 3’ endo conformation by the linkage of the 2'-oxygen and the 4'-carbon *via* a methyl bridge [135]. Furthermore, LNA anti-miRNA-192 restores the DN in mice with diabetes by reinstating the activity of the ECM inhibitor ZEB½, which is linked to reduced albuminuria and the downregulation of ECM genes [118]. These findings point to a potential therapeutic approach for DN therapy using LNA-anti-miRNAs.

### 1.18 MiRNA Inducers

The expression of miRNA is upregulated by a number of tiny compounds known as miRNA-inducing agents [104]. De-fluorinated curcumin has been shown to upregulate the level of expression of miRNA-200a in pancreatic cells [136]. The drug baicalin reduces tubular fibrosis and mesangial hypertrophy in mice by blocking the MAPK1 and TGF-β1 signaling pathways when miRNA 378 is upregulated [137]. In addition, baicalin increases the expression of miRNA-326 in HK2 cells exposed to HG, which in turn reduces the accumulation of ECM proteins by altering TGF-β/COL4/FN signaling [138].

### 1.19 MiRNA Mimic

Synthetically designed double-stranded RNA fragments known as miRNA mimics bind specifically to a target mRNA sequence to induce post-transcriptional repression [126]. The alternative strands are potentially fully or substantially complementary to the guide strand’s nucleotide sequence, which is identical to that of mature miRNA [139]. The other strand, referred to as the passenger strand, is either totally or partially corresponding to the guide strand, whereas the guide strand has a nucleotide sequence comparable to that of the mature RNA [2]. miRNA mimics can be electroporated into cells using lipid reagents. Once incorporated into the cells, the guide strand attaches to the specified genes and controls the miRNA expression [46]. The summarized novel findings on the clinical importance of miRNAs in DN for therapeutic potential based on recent studies are shown in Table **5**.

### 1.20 DN Studies of miRNA in Animal Models

Hyperglycemia decreased miR-146a while increasing IRAK1 and TRAF6 in dorsal root ganglia neurons (Fig. **4**) [39]. In diabetic mice with PN, miR-106a reduces hyperglycemia-induced oxidative stress in the DRG and sciatic nerve, leading to improved sensor and motor nerve conduction velocity [145]. A study on diabetic rats found that preventive therapy with L-arginine, ibuprofen, or both lowered miR-155 expression and nitric oxide level in the spinal cord, reducing mechanical allodynia [146]. Mir-9 levels have been identified to be elevated in the dorsal horn of the spinal cord in diabetic rats (Fig. **4**). The upregulation of CALHM1 showed a positive association, even though target scan analysis did not establish it as a probable miR-9 target [53]. P2X7 regulates ATP production and calcium levels in neuropathic pain. In diabetic mice, P2X7 regulates miR-9, resulting in upregulation of receptors and sensory hypersensitivity [147]. Further research is needed to determine how miR-9 indirectly increases CALHM1 expression, which may contribute to DN. MiRNA studies in diabetic neuropathy animal models, as shown in Table **6**.

### 1.21 Clinical Studies of miRNA in DN.

It has been investigated that the link between changes in miR-128a, miR-146a, and miR-27a and the risk of peripheral or autonomic neuropathies in patients with type 2 diabetes [160]. Mir-128a rs11888095 is linked to a higher risk of developing DN [161]. The MiR-146a variant rs-2910164 is linked to a lower risk of DN protection against the early pathophysiological condition of diabetic neuropathy [162]. Higher levels of mir1991-3p are observed in their plasma and skin samples of the lower limb [152]. MiR-1991-3p is upregulated in DN and promotes coagulation in DN by targeting the extracellular serine protease inhibitor E2 (SERPINE2), leading to tissue vascular damage associated with DN [152]. Researchers have investigated seven hundred forty-nine differentially expressed genes in nerve samples, of which 370 genes are upregulated and 379 genes are downregulated [17]. The miR-377, miR-216a, and miR-217 are involved in signaling mechanisms and inflammation [163]. Future biomarkers and treatment targets could be based on these findings on the role of DN progression. These investigations and their role in DN progression may be implemented for future biomarkers and therapeutic targets. Clinical investigations of miRNA in DN are shown in Table **7**.

## 2 PRACTICAL CHALLENGES AND LIMITATIONS

The role of miRNA in DN is crucial, yet several challenges exist in understanding its pathophysiology, diagnostic applications, and therapeutic possibilities [169]. The specific ways in which miRNAs influence diabetic neuropathy are not fully understood, and their functions at various stages of the disease require more investigation. Since miRNAs target multiple genes, it becomes challenging to determine their exact impact on diabetic neuropathy [2]. Additionally, miRNA expression differs across various tissues (such as nerves, blood, and skin), which complicates the direct relationship with the progression of neuropathy. While miRNAs hold potential as biomarkers, there is no agreement on which specific miRNAs are the most dependable for diagnosing DN [170]. Factors such as age, sex, and pre-existing health conditions can affect miRNA expression, resulting in variable diagnostic outcomes. In comparison to traditional diagnostic methods (like nerve conduction studies), miRNA-based diagnostics require further validation to ensure their accuracy and reliability [171]. Effectively delivering miRNA-based therapies to targeted nerve tissues presents a significant challenge [170]. Since miRNAs regulate multiple genes simultaneously, therapeutic interventions may have off-target effects, raising safety and efficacy concerns. Additionally, external miRNA therapies could provoke immune responses, leading to inflammation or diminished therapeutic effectiveness [170]. Most miRNA-based treatments are still in the early stages of research, and extensive human trials are necessary to establish their safety and effectiveness [17]. The development of miRNA therapy is complex, making it challenging to obtain regulatory approval for clinical applications [172]. Additionally, miRNA-based diagnostics and treatments can be costly, which may hinder their adoption in clinical settings. The long-term effects of miRNA modulation on patients with diabetic neuropathy remain unclear. Although miRNAs show potential for enhancing our understanding, diagnosis, and treatment of diabetic neuropathy, numerous scientific, technical, and regulatory hurdles need to be overcome before they can be fully implemented in clinical practice [173]. It is anticipated that upcoming investigations will focus on standardizing profiles of miRNA biomarkers that demonstrate sensitivity and diagnoses accuracy, without significant discrimination related to miRNA [174]. Artificial intelligence may enhance the precision of miRNA-based diagnostics by pattern recognition of miRNA expression in relevant disease-specific datasets [175]. For instance, treatment strategies may emerge that utilize miRNA mimics (to restore beneficial miRNAs), and antagonism (to inhibit bad miRNAs) that will likely progress into clinical trials [139]. Delivery systems based on nanoparticles may improve stability and effectiveness. Advancements in CRISPR-based gene editing in the future may enable diseases to be treated with greater accuracy by altering miRNA expression, providing a therapeutic approach for the longer duration [176]. Continued research and clinical validation are crucial to address these issues. The translational challenges in using miRNA as biomarkers or therapeutic targets for DN are shown in Table **8**.

## CONCLUSION

This review highlights recent advances on the clinical importance of miRNAs in diabetic neuropathy, emphasizing their roles in pathophysiology, diagnosis, and potential therapies. While preclinical studies are promising, further clinical validation is essential to overcome current translational challenges. In preclinical investigations, miRNA-based diagnosis and therapeutic approaches have shown potential for clinical applications, but at the same time, there still remain various challenges to be addressed for their implementation in clinical practice, including potential toxicities, specificity, and instability. A network of miRNAs will likely be able to characterize DN more thoroughly and accurately. Research on the methods of miRNA control in the development of DN is limited. The majority of investigations on miRNAs are carried out using animal models; however, for the clinical implications of miRNA in diagnosis and treatment, further investigations are required. In the animal model of DN, recent preclinical research employing exogenous double-stranded mimics or single-stranded antisense RNA has produced encouraging outcomes. miRNAs have great potential to improve our understanding, diagnosis, and treatment of DN. Ongoing research and technological innovations are anticipated to tackle current challenges and boost their clinical use. Advanced bioinformatics will aid in pinpointing the key miRNAs involved in the development of DN, thereby enhancing our grasp of their regulatory networks. Future investigations may reveal how miRNA interacts with various signaling pathways, such as oxidative stress, inflammation, and neurodegeneration, leading to the creation of more targeted therapeutic strategies. Exploring the relationship between miRNA and epigenetic modifications in DN could pave the way for new methods of disease management. The creation of blood and urine-based miRNA panels could facilitate early and non-invasive detection of DN. Future studies are likely to focus on standardizing miRNA biomarker profiles that exhibit high sensitivity, as well as miRNA-based diagnosis. Artificial intelligence can improve the accuracy of miRNA-based diagnostics by analyzing extensive datasets and recognizing disease-specific miRNA expression patterns. Therapeutic approaches utilizing miRNA mimics (to restore beneficial miRNAs) and antagonism (to suppress detrimental miRNAs) are expected to advance into clinical trials. Treatment based on miRNA could be combined with current pharmacological interventions, such as antioxidants and anti-inflammatory agents, to enhance neuroprotection in DN. Standard quality control procedures must be considered in order to ensure the consistency of the exosomal product and the content of its miRNA delivery.

## Figures and Tables

**Fig. (1):**
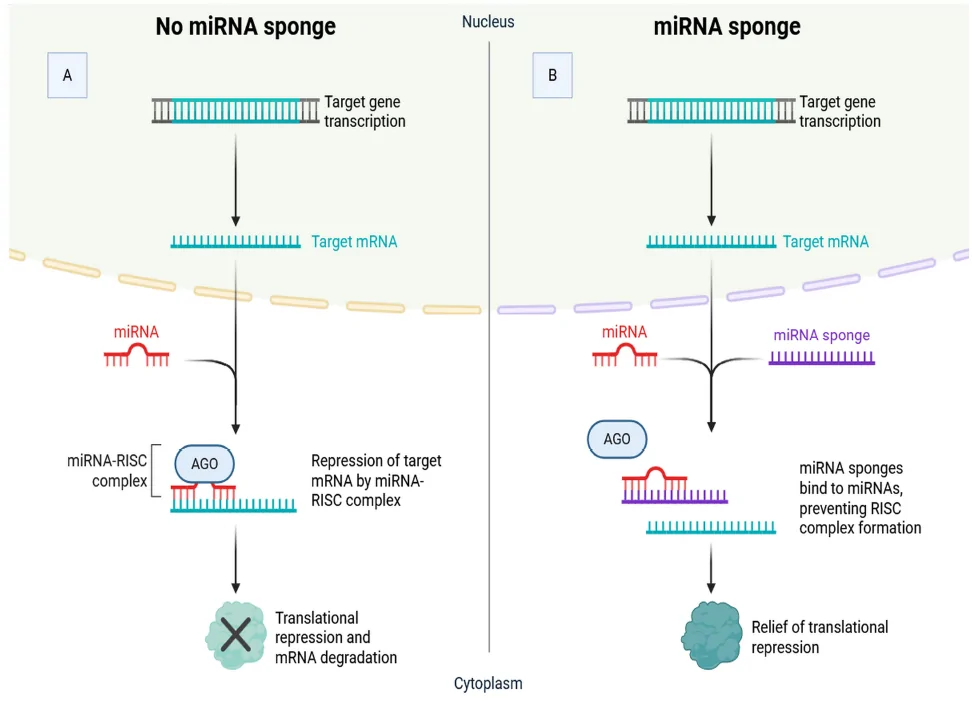
Mechanism of miRNA sponge-mediated regulation of gene expression. (**A**) In the absence of a miRNA sponge, microRNAs (miRNAs) bind to their target mRNAs by forming a miRNA-induced silencing complex (miRNA-RISC), which includes Argonaute (AGO) proteins. This leads to repression of target mRNA through translational inhibition and/or mRNA degradation. (**B**) In the presence of a miRNA sponge, miRNAs are sequestered by sponge transcripts, preventing their interaction with the target mRNA and the formation of the miRNA-RISC complex. This results in relief of translational repression and allows for target gene expression.

**Fig. (2):**
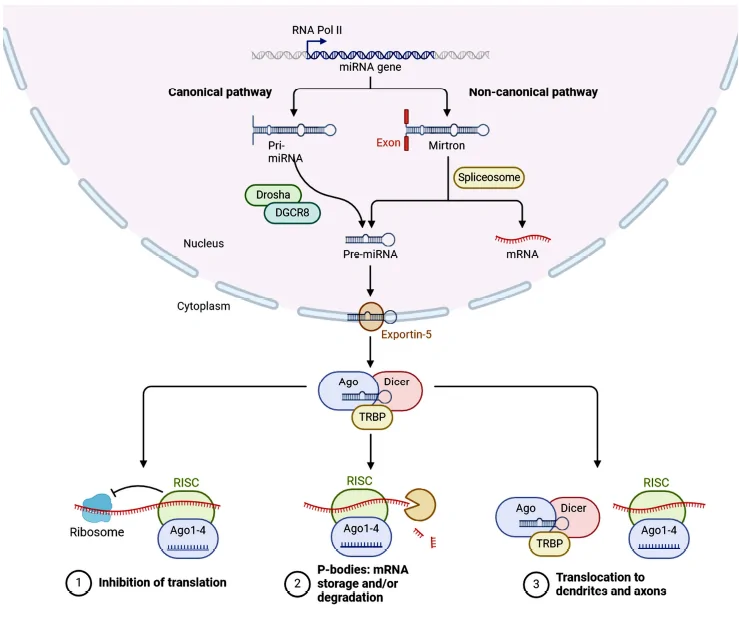
MiRNA processing mechanisms in the diabetic neuropathy: (1) Inhibition of translation, (2) Mechanism of mRNA storage and degradation, (3) mechanism of translocation to dendrites and axons.

**Fig. (3):**
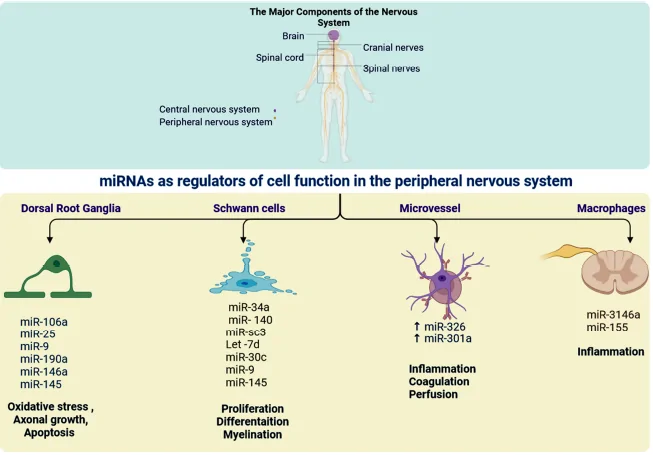
miRNAs are key post-transcriptional regulators in the peripheral nervous system, influencing various cellular processes like oxidative stress response, axonal regeneration, and inflammation. Their dysregulation has been implicated in diabetic neuropathy, making them promising therapeutic targets. MiRNAs as regulators of cell function in the peripheral nervous system play a crucial role in modulating oxidative stress, axonal growth, apoptosis, proliferation, differentiation, myelination, and perfusion. Their involvement in processes such as neuronal development, response to injury, and inflammation highlights their potential as therapeutic targets for diabetic neuropathy.

**Fig. (4):**
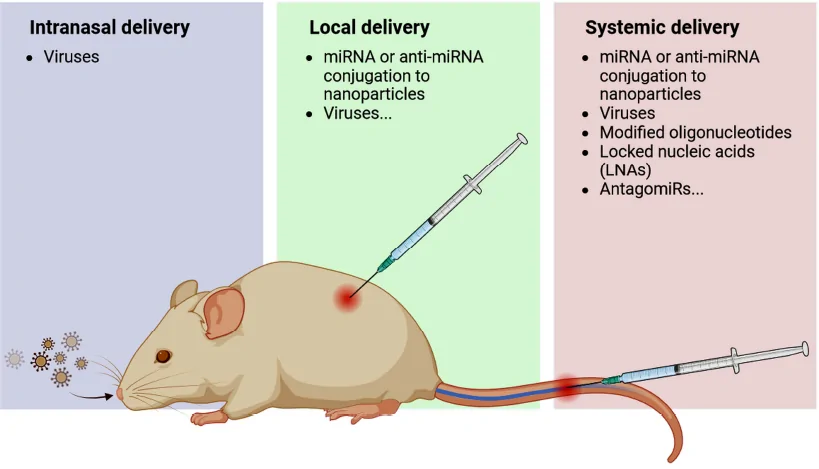
MiRNA targeting approaches in animal models.

**Table 1:** Studied miRNA in human diabetic neuropathy.

**S. No.**	**Type of miRNA**	**Origin**	**Type of Expression**	**References**
1	miR-618, miR-518d-3p	Serum sample	Up-regulated	[38]
2	miR-146a	Dorsal Root Ganglion (DRG)	Up-regulated	[39]
3	miR-155	White blood cells, sural nerve, lower leg skin	Up-regulated	[40]
4	miR-199a-3p	Skin biopsies	Up-regulated	[41]
5	miR-216a	Nerve tissue sample	Up-regulated	[42]
6	miR-21	White blood cells, sural nerve	Up-regulated	[43]
7	miR-49a-3p with rs3746444 SNP	Peripheral blood	Up-regulated	[2]
8	miR-203, miR-181-a1, miR-541	Spinal dorsal horn	Down-regulated	[2]
9	miR-377	Nerve tissue sample	Up-regulated	[44]
10	miR-21, miR-29a/b/c, miR-192	DRG	Up-regulated	[2]
11	miR-199a-3p	Plasma	Up-regulated	[41]
12	miR-146	White blood cells, blood mono-nuclear cells	Down-regulated	-
13	miR-125a-5p, miR-873-5p, miR-99-5p	Blood mono-nuclear cells	Up-regulated	[2]
14	miR-217	Nerve tissue sample	Up-regulated	[45]

**Table 2:** Novel findings on the clinical importance of miRNAs in Diabetic Neuropathy (DN) pathophysiology based on recent studies.

**miRNA**	**Target Pathway**	**Role in Pathophysiology**	**Key Findings**	**Reference**
miR-21	NF-κB, PTEN, TGF-β	Promotes inflammation and fibrosis	Upregulated in DN, enhances fibrosis *via* TGF-β and suppresses PTEN, leading to nerve damage	[14]
miR-146a	IRAK1, TRAF6, NF-κB	Anti-inflammatory, neuroprotective	Downregulated in DN; miRNA-146a mimics improve nerve conduction and reduce inflammation	[46]
miR-29b	TGF-β/SMAD3	Protects against fibrosis and apoptosis	Downregulation in DN leads to increased fibrosis and neuronal apoptosis	[47]
miR-217	PI3K/Akt	Regulates neuronal survival and vascular dysfunction	Upregulated in DN, contributes to glomerular hypertrophy and endothelial damage	[45]
miR-125b	VDR, Apoptotic Pathways	Enhances Schwann cell apoptosis	Upregulation leads to impaired nerve repair and worsens oxidative stress	[48]
miR-9	CALHM1, ATP-P2X7R	Modulates neuropathic pain	Upregulated in DN, enhances pain signaling *via* the ATP-P2X7R pathway	[49]
miR-155	PI3K/Akt/mTOR, Nrf2	Regulates oxidative stress and inflammation	Increased levels contribute to neuroinflammation and reduced autophagy	[40]
miR-199a-3p	SerpinE2, MMP-13	Involved in vascular dysfunction	Promotes coagulation and endothelial dysfunction, worsening DN progression	[41]

**Table 3:** Novel findings on the clinical importance of miRNAs in Diabetic Neuropathy (DN) diagnosis based on recent studies.

**miRNA**	**Sample Type**	**Expression in DN**	**Diagnostic Significance**	**References**
miR-146a	Blood, WBCs	Downregulated	Potential biomarker for early DN detection; associated with reduced inflammation and nerve protection	[84]
miR-21	Plasma, Nerve Biopsy	Upregulated	Correlates with nerve fibrosis and inflammation; potential marker for DN progression	[85]
miR-29b	Serum, DRG	Downregulated	Associated with increased fibrosis and nerve degeneration; a diagnostic marker for DN severity	[2]
miR-499a-3p	Peripheral Blood	Upregulated	Linked to genetic predisposition for DN; potential biomarker for DN susceptibility	[86]
miR-199a-3p	Skin Biopsy, Plasma	Upregulated	Promotes vascular dysfunction and coagulation; potential indicator of DN-related microvascular complications	[41]
miR-125b-5p	White Blood Cells	Upregulated	Correlates with Schwann cell apoptosis and impaired nerve regeneration	[87]
miR-155	Peripheral Blood, Sural Nerve	Upregulated	Involved in neuroinflammation; potential biomarker for pain-related DN	[88]
miR-133a-3p	Serum	Downregulated	Associated with pain modulation; potential diagnostic tool for neuropathic pain in DN [89]	[90]
miR-126	Serum	Downregulated	Linked to endothelial dysfunction and vascular complications in DN	[89]
miR-206	Skin and Nerve Tissue	Downregulated	Associated with neuronal apoptosis; potential biomarker for nerve degeneration in DN	[91]

**Table 4:** Pathophysiological processes in Diabetic Neuropathy (DN) and their interactions with specific molecular mechanisms.

**Pathophysiological Process**	**Key Molecular Mechanisms**	**miRNAs Involved**	**Impact on DN**	**Reference**
Neuroinflammation	NF-κB, IRAK1, TRAF6, TNF-α, IL-6	miR-146a, miR-155, miR-21	miR-146a reduces inflammation, while miR-155 and miR-21 promote pro-inflammatory cytokine release	[125]
Oxidative stress	Nrf2, ROS, 12/15-LOX, SOD	miR-155, miR-160a-5p, miR-21	miR-155 inhibits Nrf2, increasing oxidative stress; miR-160a-5p downregulates 12/15-LOX to reduce ROS	[126]
Fibrosis & Extracellular Matrix (ECM) accumulation	TGF-β/SMAD3, COL1A1, COL1A2	miR-29b, miR-21, miR-192	miR-29b prevents fibrosis by inhibiting TGF-β, whereas miR-21 and miR-192 promote ECM deposition	[127]
Schwann cell dysfunction	PI3K/Akt, Krox20, Notch1	miR-34a, miR-140, Let-7d	miR-34a impairs myelination, miR-140 affects Krox20, Let-7d reduces Schwann cell proliferation	[28]
Neurovascular dysfunction	VEGF, HIF-1α, eNOS	miR-126, miR-199a-3p, miR-217	miR-126 enhances endothelial repair, while miR-199a-3p and miR-217 promote vascular dysfunction	[128]
Neuropathic pain sensitization	P2X7R, Nav1.3, SLC17A6	miR-9, miR-133a-3p, miR-190a-5p	miR-9 enhances P2X7R-mediated pain signaling, miR-133a-3p downregulates Nav1.3, reducing pain	[53]
Apoptosis & neuronal degeneration	BAX/BCL2, SIRT1, Caspase Pathways	miR-125b, miR-206, miR-217	miR-125b induces Schwann cell apoptosis, miR-206 prevents neuronal degeneration	[129]
Metabolic dysregulation	Insulin/IGF-1, AMPK, GLUT4	miR-29c, miR-214, miR-132	miR-29c impairs glucose metabolism, while miR-214 and miR-132 regulate insulin signaling	[130]

**Table 5:** Clinical importance of miRNAs in DN for therapeutic potential based on recent studies.

**miRNA**	**Target Pathway**	**Therapeutic Role**	**Potential Intervention**	**Reference**
miR-21	PTEN, TGF-β, NF-κB	Promotes fibrosis and inflammation	miR-21 inhibitors may reduce nerve damage and improve neuroprotection	[43]
miR-146a	IRAK1, TRAF6, NF-κB	Anti-inflammatory, neuroprotective	miR-146a mimics reduce inflammation and improve nerve conduction in DN models	[39]
miR-29b	TGF-β/SMAD3	Protects against fibrosis and apoptosis	miR-29b mimics prevent nerve degeneration and improve neuronal survival	[140]
miR-155	PI3K/Akt/mTOR, Nrf2	Regulates oxidative stress and inflammation	miR-155 inhibitors reduce neuroinflammation and oxidative stress	[141]
miR-499a-3p	PI3K, NF-κB	Modulates pain sensitivity and nerve regeneration	Targeting miR-499a may help manage neuropathic pain and nerve repair	[142]
miR-133a-3p	TLR4, Nav1.3	Pain modulation	miR-133a overexpression may reduce pain hypersensitivity in DN	[143]
miR-199a-3p	SerpinE2, MMP-13	Reduces vascular dysfunction	miR-199a inhibitors could prevent DN-related vascular complications	[41]
miR-126	VEGF, Endothelial Repair	Enhances vascular protection	miR-126 therapy may improve blood flow and nerve regeneration	[144]
miR-214	PTEN, NF-κB	Anti-inflammatory, promotes nerve healing	miR-214 mimics may restore nerve function and reduce inflammation	[14]
miR-206	RAGE, Apoptosis Pathways	Prevents neuronal degeneration	miR-206 therapy could slow DN progression by protecting neurons	[91]

**Table 6:** MiRNA studies in diabetic neuropathy animal models.

**S. No.**	**Type of sample**	**Type of miRNA**	**Target**	**regulations**	**Findings**	**References**
1	Blood samples	miRNA-155	PI3KCA	Downregulations	Promoting cellular autophagy while inhibiting apoptosis by suppressing PI3K/Akt/mTOR signaling pathway	[110]
2	SN rat	miR-133a-3p	TLR4	Upregulations	Alleviating the nerve symptoms by regulating the expression of Nav1.3	[148]
3	Serum exosomes rat	miR-221-3p	SOCS3	Upregulations	Inhibits the inflammation by suppressing the Nf-κB signaling pathway	[149]
4	SN -rat model	miRNA-155	Nrf2	Upregulations	Inhibits cellular autophagy by regulating the expression of Nrf2, and plays an important role in inflammation and oxidative stress	[150]
5	DRG mice	miR-146a-5p	IRAK1, TRAF6	Downregulations	Affects the inflammation by downregulating the polarization of macrophages to M1	[151]
6	DRG mice-SN rat	miR-214-3p	SCN 3A	Downregulations	Inhibits the inflammation by suppressing the NF-κB signaling pathway	[152]
7	PC12 cell line	miRNA-125b-5p	VDR	Upregulations	Exacerbates the cellular apoptosis by decreasing the expression of VDR	[129]
8	DRG mice	miR-590-3p	RAP1A	Downregulations	Ameliorating inflammation by inhibiting the T-cell infiltration	[153]
9	DRG mice	miRNA-160a-5p	12/15 LOX	Downregulation	Inhibits oxidative stress by reducing the expression of 12/15 Lox	[154]
10	SN rat	miRNA-7a-5p	VDAC1	Downregulation	Inhibits the activation of JNK/C-Jun and alleviates apoptosis	-
11	Nerve tissue	miRNA-130a-5p	DNMT1	Downregulation	Inhibits cellular apoptosis by regulating the DNMT1/NRF2/HIF1α axis	[155]
12	DRG/SN mice	miR-206	RAGE	Downregulation	Increases the expression of RAGE expression	[156]
13	DRG /SN/FP mice	miRNA-34a-5p	FOXP2	Upregulations	Inhibits the axon growth	[157]
14	DRG /SN rat	miRNA-34a-5p	SIRT2	Upregulations	Exacerbates the oxidative stress by inhibiting the expression of SIRT2	[158]
15	SN rat	Let-7i	Unknown	Downregulations	Promote axonal growth of DRG neurons	[2]
16	DRG/SN/FP mice	miR-29b	TGF-β/Smad3 pathway	Downregulation	Protect DRG neurons against apoptosis	[159]
17	DRG/SN/FP mice	miR-29c	PRCK1	Downregulation	Inhibit the axon growth	[58]
18	DRG mice	miRNA-199a	SERPINE2, MMP-13	Upregulations	Promote coagulation	[41]

**Table 7:** Clinical studies of miRNA in diabetic neuropathy.

**S. No.**	**Clinical condition of the patient**	**Sample type and**	**Clinical condition of the patient**	**Regulations of miRNA**	**Clinical outcome correlation with pain process pathways**	**References**
1	23 participants with neuropathy of diabetes	Plasma and sural nerve biopsies	23 participants with neuropathy of diabetes	Reduced expression in miR-101, increased expression in miR-132	Reduced miR-101, in neuropathic pain patients versus controls, miRNA-101 expression is negatively correlated with KPNB1	[164]
2	Blood samples 40 participants with diabetes without neuropathy	Blood samples	Blood samples 40 participants with diabetes without neuropathy	Downregulation of miR-1-3p	Significant expression reduced in miR-1-3p diabetic neuropathic group compared to the type 2 diabetes mellitus group	[165]
3	141 participants with diabetic neuropathy	WBC and sural nerve biopsies	141 participants with diabetic neuropathy	Increase in miRN-132-3p	Increased the expression level of miRN-132-3p by 2.6 fold	[166]
4	40 participants with diabetes with/without neuropathy and 10 healthy controls	Blood samples	40 participants with diabetes with/without neuropathy and 10 healthy controls	Downregulation of miR-19b-3p, and miR-125a-5p	Lower expression of miR-19b-3p and miR-125a-5p in the diabetic neuropathy group compared with the healthy group.	[165]
5	20 participants with neuropathy of various etiology	Peripheral blood mononuclear cells/CD4+T cells	20 participants with neuropathy of various etiology.	Increase in miRN-124a and miR-155 reduced expression of in PBMC using real-time PCR	Increased expression of miR-124a and miR-155 reduced the expression of SIRT1 in neuropathic patients compared with controls	[167]
6	40 participants with diabetes with/without neuropathy and 10 healthy controls	Blood samples	40 participants with diabetes with/without neuropathy and 10 healthy controls	Downregulation of miR-183-5p and miR-33-3p	Expression of miRNA-183-5p downregulated in diabetic neuropathy compared to the control group; Expression of miRNA-183-5p and miRNA-33-3p Downregulated in diabetic neuropathy compared to without neuropathic group	[168]

**Table 8:** Translational challenges in using miRNA as biomarkers or therapeutic targets for Diabetic Neuropathy (DN).

**Challenge**	**Description**	**Implications for DN Research & Treatment**	**Potential Solutions**	**Reference**
Variability in miRNA expression across tissues	miRNA levels vary between blood, nerve, skin, and other tissues	Reduces the reliability of miRNA biomarkers for DN diagnosis and monitoring	Standardization of tissue-specific miRNA panels and normalization strategies	[177]
Demographic and patient-specific variability	Age, sex, ethnicity, and comorbidities (*e.g.*, diabetes duration, metabolic status) influence miRNA profiles	Results in inconsistent biomarker performance across different populations	Large-scale, multi-cohort validation studies across diverse demographics	[17]
Off-target effects in miRNA-based therapies	miRNAs regulate multiple genes and pathways, leading to unintended effects	Potential adverse outcomes, including disruption of essential physiological pathways	Use of highly specific miRNA inhibitors, sponges, or CRISPR-based editing for precision targeting	[178]
Stability and degradation in circulation	Circulating miRNAs are susceptible to enzymatic degradation (*e.g.*, RNases) and environmental factors	Reduces the sensitivity and specificity of miRNA-based diagnostics	Encapsulation in exosomes, liposomes, or nanoparticles to improve stability	[179]
Challenges in delivery to peripheral nerves	Limited ability to cross the Blood-Brain Barrier (BNB) and reach target tissues	Reduces the therapeutic efficacy of miRNA-based treatments for DN	Development of nanoparticle, lipid-based, and exosome-mediated delivery systems	[180]
Lack of standardized reference ranges	No universally accepted miRNA expression levels for DN	Creates challenges in clinical interpretation and diagnostic reproducibility	Establishing standardized cutoff values through large clinical trials	[177]
Potential immune responses to miRNA therapies	Exogenous miRNA delivery may trigger immune activation and unwanted inflammatory responses	Limits safety and applicability in therapeutic settings	Optimization of chemical modifications, delivery vehicles, and immunogenicity testing	[181]
Ethical and regulatory barriers	CRISPR-based miRNA editing raises concerns about unintended genetic alterations and long-term safety	Delays in regulatory approval and clinical translation	Comprehensive preclinical safety evaluations and adherence to regulatory guidelines	[182]
High cost and scalability issues	miRNA-based diagnostics and therapeutics require advanced molecular technologies	Limits the accessibility and feasibility of large-scale implementation	Development of cost-effective, scalable miRNA detection and delivery platforms	[183]

## LIST OF ABBREVIATIONS

DN: Diabetic Neuropathy
AGEs: Advanced Glycation End Products
PKC: Protein Kinase C
miRNA-RISC: miRNA-induced Silencing Complex
AGO: Argonaute
SD: Sorbitol Dehydrogenase
AGEs: Advanced Glycation End Products
HIF-1α: Hypoxia-Inducible Factor
MNCV: Motornerve Conduction Velocity
CFA: Complete Freund's Adjuvant
ZEB2: E Box Binding Homeobox 2

## References

[1] Zaino B., Goel R., Devaragudi S., Prakash A., Vaghamashi Y., Sethi Y., Patel N., Kaka N. (2023). Diabetic neuropathy: Pathogenesis and evolving principles of management.. Dis. Mon..

[2] Fan B., Chopp M., Zhang Z.G., Liu X.S. (2020). Emerging roles of micrornas as biomarkers and therapeutic targets for diabetic neuropathy.. Front. Neurol..

[3] Ranganathan K., Sivasankar V. (2014). MicroRNAs - Biology and clinical applications.. J. Oral Maxillofac. Pathol..

[4] Lee Y., Ahn C., Han J., Choi H., Kim J., Yim J., Lee J., Provost P., Rådmark O., Kim S., Kim V.N. (2003). The nuclear RNase III Drosha initiates microRNA processing.. Nature.

[5] Simeoli R., Fierabracci A. (2019). Insights into the role of micrornas in the onset and development of diabetic neuropathy.. Int. J. Mol. Sci..

[6] Samsu N. (2021). Diabetic Nephropathy: Challenges in pathogenesis, diagnosis, and treatment.. BioMed Res. Int..

[7] Lv Y., Yao X., Li X., Ouyang Y., Fan C., Qian Y. (2024). Cell metabolism pathways involved in the pathophysiological changes of diabetic peripheral neuropathy.. Neural Regen. Res..

[8] Ratan Y., Rajput A., Pareek A., Pareek A., Kaur R., Sonia S., Kumar R., Singh G. (2024). Recent advances in biomolecular patho-mechanistic pathways behind the development and progression of diabetic neuropathy.. Biomedicines.

[9] Pang L., Lian X., Liu H., Zhang Y., Li Q., Cai Y., Ma H., Yu X. (2020). Understanding diabetic neuropathy: Focus on oxidative stress.. Oxid. Med. Cell. Longev..

[10] Szostak J., Gorący A., Durys D., Dec P., Modrzejewski A., Pawlik A. (2023). The role of microrna in the pathogenesis of diabetic nephropathy.. Int. J. Mol. Sci..

[11] Massaro J.D., Polli C.D., Costa e Silva M., Alves C.C., Passos G.A., Sakamoto-Hojo E.T., Rodrigues de Holanda Miranda W., Bispo Cezar N.J., Rassi D.M., Crispim F., Dib S.A., Foss-Freitas M.C., Pinheiro D.G., Donadi E.A. (2019). Post-transcriptional markers associated with clinical complications in Type 1 and Type 2 diabetes mellitus.. Mol. Cell. Endocrinol..

[12] Kenyon J.D., Sergeeva O., Somoza R.A., Li M., Caplan A.I., Khalil A.M., Lee Z. (2019). Analysis of -5p and -3p Strands of miR-145 and miR-140 During Mesenchymal Stem Cell Chondrogenic Differentiation.. Tissue Eng. Part A.

[13] Sebastiani G., Guarino E., Grieco G.E., Formichi C., Delli Poggi C., Ceccarelli E., Dotta F. (2017). Circulating microRNA (miRNA) expression profiling in plasma of patients with gestational diabetes mellitus reveals upregulation of miRNA miR-330-3p.. Front. Endocrinol..

[14] Tang J., Yao D., Yan H., Chen X., Wang L., Zhan H. (2019). The role of micrornas in the pathogenesis of diabetic nephropathy.. Int. J. Endocrinol..

[15] Zhu J., Hu Z., Luo Y., Liu Y., Luo W., Du X., Luo Z., Hu J., Peng S. (2024). Diabetic peripheral neuropathy: Pathogenetic mechanisms and treatment.. Front. Endocrinol..

[16] Galiero R., Caturano A., Vetrano E., Beccia D., Brin C., Alfano M., Di Salvo J., Epifani R., Piacevole A., Tagliaferri G., Rocco M., Iadicicco I., Docimo G., Rinaldi L., Sardu C., Salvatore T., Marfella R., Sasso F.C. (2023). Peripheral neuropathy in diabetes mellitus: Pathogenetic mechanisms and diagnostic options.. Int. J. Mol. Sci..

[17] Yang Y., Zhao B., Wang Y., Lan H., Liu X., Hu Y., Cao P. (2025). Diabetic neuropathy: Cutting-edge research and future directions.. Signal Transduct. Target. Ther..

[18] Gutierrez J., Palma J.A., Kaufmann H. (2020). Acute sensory and autonomic neuronopathy: A devastating disorder affecting sensory and autonomic ganglia.. Semin. Neurol..

[19] Vincent A.M., Russell J.W., Low P., Feldman E.L. (2004). Oxidative stress in the pathogenesis of diabetic neuropathy.. Endocr. Rev..

[20] Grote C.W., Wright D.E. (2016). A role for insulin in diabetic neuropathy.. Front. Neurosci..

[21] Niimi N., Yako H., Takaku S., Chung S.K., Sango K. (2021). Aldose reductase and the polyol pathway in schwann cells: Old and new problems.. Int. J. Mol. Sci..

[22] Garg S.S., Gupta J. (2022). Polyol pathway and redox balance in diabetes.. Pharmacol. Res..

[23] Thorne C.A., Grey A.C., Lim J.C., Donaldson P.J. (2024). The synergistic effects of polyol pathway-induced oxidative and osmotic stress in the aetiology of diabetic cataracts.. Int. J. Mol. Sci..

[24] Kilanczyk E., Saraswat Ohri S., Whittemore S.R., Hetman M. (2016). Antioxidant protection of nadph-depleted oligodendrocyte precursor cells is dependent on supply of reduced glutathione.. ASN Neuro.

[25] Byun K., Yoo Y., Son M., Lee J., Jeong G.B., Park Y.M., Salekdeh G.H., Lee B. (2017). Advanced glycation end-products produced systemically and by macrophages: A common contributor to inflammation and degenerative diseases.. Pharmacol. Ther..

[26] Sifuentes-Franco S., Pacheco-Moisés F.P., Rodríguez-Carrizalez A.D., Miranda-Díaz A.G. (2017). The role of oxidative stress, mitochondrial function, and autophagy in diabetic polyneuropathy.. J. Diabetes Res..

[27] Pitocco D., Tesauro M., Alessandro R., Ghirlanda G., Cardillo C. (2013). Oxidative stress in diabetes: Implications for vascular and other complications.. Int. J. Mol. Sci..

[28] Wu L., Wang X.J., Luo X., Zhang J., Zhao X., Chen Q. (2024). Diabetic peripheral neuropathy based on Schwann cell injury: Mechanisms of cell death regulation and therapeutic perspectives.. Front. Endocrinol..

[29] Koya D., King G.L. (1998). Protein kinase C activation and the development of diabetic complications.. Diabetes.

[30] Gonçalves N.P., Vægter C.B., Andersen H., Østergaard L., Calcutt N.A., Jensen T.S. (2017). Schwann cell interactions with axons and microvessels in diabetic neuropathy.. Nat. Rev. Neurol..

[31] Menini S., Iacobini C., Vitale M., Pugliese G. (2020). The inflammasome in chronic complications of diabetes and related metabolic disorders.. Cells.

[32] Nashtahosseini Z., Eslami M., Paraandavaji E., Haraj A., Dowlat B.F., Hosseinzadeh E., Oksenych V., Naderian R. (2025). Cytokine signaling in diabetic neuropathy: A key player in peripheral nerve damage.. Biomedicines.

[33] Wang Q., Ye Y., Yang L., Xiao L., Liu J., Zhang W., Du G. (2024). Painful diabetic neuropathy: The role of ion channels.. Biomed. Pharmacother..

[34] Song Z., Fu D.T., Chan Y.S., Leung S., Chung S.S., Chung S.K. (2003). Transgenic mice overexpressing aldose reductase in Schwann cells show more severe nerve conduction velocity deficit and oxidative stress under hyperglycemic stress.. Mol. Cell. Neurosci..

[35] Hao W., Tashiro S., Hasegawa T., Sato Y., Kobayashi T., Tando T., Katsuyama E., Fujie A., Watanabe R., Morita M., Miyamoto K., Morioka H., Nakamura M., Matsumoto M., Amizuka N., Toyama Y., Miyamoto T. (2015). Hyperglycemia Promotes Schwann Cell De-differentiation and De-myelination *via* Sorbitol Accumulation and Igf1 Protein Down-regulation.. J. Biol. Chem..

[36] Drel V.R., Pacher P., Stevens M.J., Obrosova I.G. (2006). Aldose reductase inhibition counteracts nitrosative stress and poly(ADP-ribose) polymerase activation in diabetic rat kidney and high-glucose-exposed human mesangial cells.. Free Radic. Biol. Med..

[37] Feldman E.L., Nave K.A., Jensen T.S., Bennett D.L.H. (2017). New horizons in diabetic neuropathy: Mechanisms, bioenergetics, and pain.. Neuron.

[38] Santos-Bezerra D.P., Santos A.S., Guimarães G.C., Admoni S.N., Perez R.V., Machado C.G., Pelaes T.S., Passarelli M., Machado U.F., Queiroz M.S., da Silva M.E.R., Correa-Giannella M.L. (2019). Micro-RNAs 518d-3p and 618 Are Upregulated in Individuals With Type 1 Diabetes With Multiple Microvascular Complications.. Front. Endocrinol..

[39] Wang L., Chopp M., Szalad A., Zhang Y., Wang X., Zhang R.L., Liu X.S., Jia L., Zhang Z.G. (2014). The role of miR-146a in dorsal root ganglia neurons of experimental diabetic peripheral neuropathy.. Neuroscience.

[40] Ji H., Lu Y., Liu G., Zhao X., Xu M., Chen M. (2024). Role of decreased expression of mir-155 and mir-146a in peripheral blood of type 2 diabetes mellitus patients with diabetic peripheral neuropathy.. Diabetes Metab. Syndr. Obes..

[41] Li Y.B., Wu Q., Liu J., Fan Y.Z., Yu K.F., Cai Y. (2017). miR-199a-3p is involved in the pathogenesis and progression of diabetic neuropathy through downregulation of SerpinE2.. Mol. Med. Rep..

[42] Xia H., Ooi L.L.P.J., Hui K.M. (2013). MicroRNA-216a/217-induced epithelial-mesenchymal transition targets PTEN and SMAD7 to promote drug resistance and recurrence of liver cancer.. Hepatology.

[43] Liu Y., Tian M., Yang Y., Li H., Zhao T., Zhu J., Mou F., Cui G., Guo H., Shao S. (2022). Schwann cells-derived exosomal miR-21 participates in high glucose regulation of neurite outgrowth.. iScience.

[44] Wang Q., Wang Y., Minto A.W., Wang J., Shi Q., Li X., Quigg R.J. (2008). MicroRNA-377 is up-regulated and can lead to increased fibronectin production in diabetic nephropathy.. FASEB J..

[45] Sun J., Li Z.P., Zhang R.Q., Zhang H.M. (2017). Repression of miR-217 protects against high glucose-induced podocyte injury and insulin resistance by restoring PTEN-mediated autophagy pathway.. Biochem. Biophys. Res. Commun..

[46] Liu X.S., Fan B., Szalad A., Jia L., Wang L., Wang X., Pan W., Zhang L., Zhang R., Hu J., Zhang X.M., Chopp M., Zhang Z.G. (2017). MicroRNA-146a mimics reduce the peripheral neuropathy in type 2 diabetic mice.. Diabetes.

[47] Chen H.Y., Zhong X., Huang X.R., Meng X.M., You Y., Chung A.C.K., Lan H.Y. (2014). MicroRNA-29b inhibits diabetic nephropathy in db/db mice.. Mol. Ther..

[48] Hou X., Xu F., Zhang C., Shuai J., Huang Z., Liang Y., Xu X. (2020). Dexmedetomidine exerts neuroprotective effects during high glucose-induced neural injury by inhibiting miR-125b.. Biosci. Rep..

[49] Sun Q., Zeng J., Liu Y., Chen J., Zeng Q.C., Chen Y.Q., Tu L.L., Chen P., Yang F., Zhang M. (2020). microRNA-9 and -29a regulate the progression of diabetic peripheral neuropathy *via* ISL1-mediated sonic hedgehog signaling pathway.. Aging.

[50] Donate-Correa J., Luis-Rodríguez D., Martín-Núñez E., Tagua V.G., Hernández-Carballo C., Ferri C., Rodríguez-Rodríguez A.E., Mora-Fernández C., Navarro-González J.F. (2020). Inflammatory targets in diabetic nephropathy.. J. Clin. Med..

[51] Stoian A., Muntean C., Babă D.F., Manea A., Dénes L., Simon-Szabó Z., Kosovski I.B., Nemes-Nagy E., Gliga F.I., Stoian M. (2024). Update on biomarkers of chronic inflammatory processes underlying diabetic neuropathy.. Int. J. Mol. Sci..

[52] Barutta F., Bellini S., Mastrocola R., Bruno G., Gruden G. (2018). MicroRNA and microvascular complications of diabetes.. Int. J. Endocrinol..

[53] Liu W., Ao Q., Guo Q., He W., Peng L., Jiang J., Hu X. (2017). miR-9 Mediates CALHM1-activated ATP-P2X7R signal in painful diabetic neuropathy rats.. Mol. Neurobiol..

[54] Duan Z., Zhang J., Li J., Pang X., Wang H. (2020). Inhibition of microRNA-155 reduces neuropathic pain during chemotherapeutic bortezomib *via* engagement of neuroinflammation.. Front. Oncol..

[55] Yang D., Yang Q., Wei X., Liu Y., Ma D., Li J., Wan Y., Luo Y. (2017). The role of miR-190a-5p contributes to diabetic neuropathic pain *via* targeting SLC17A6.. J. Pain Res..

[56] Karnati H.K., Panigrahi M.K., Gutti R.K., Greig N.H., Tamargo I.A. (2015). miRNAs: Key Players in neurodegenerative disorders and epilepsy.. J. Alzheimers Dis..

[57] Zhang X., Gong X., Han S., Zhang Y. (2014). MiR-29b protects dorsal root ganglia neurons from diabetic rat.. Cell Biochem. Biophys..

[58] Jia L., Wang L., Chopp M., Li C., Zhang Y., Szalad A., Zhang Z.G. (2018). MiR-29c/PRKCI regulates axonal growth of dorsal root ganglia neurons under hyperglycemia.. Mol. Neurobiol..

[59] Corradi E., Baudet M.L. (2020). In the right place at the right time: Mirnas as key regulators in developing axons.. Int. J. Mol. Sci..

[60] Jia L., Wang L., Chopp M., Zhang Y., Szalad A., Zhang Z.G. (2016). MicroRNA 146a locally mediates distal axonal growth of dorsal root ganglia neurons under high glucose and sildenafil conditions.. Neuroscience.

[61] Sohn E.J., Park H.T. (2018). MicroRNA mediated regulation of schwann cell migration and proliferation in peripheral nerve injury.. BioMed Res. Int..

[62] Verrier J.D., Semple-Rowland S., Madorsky I., Papin J.E., Notterpek L. (2010). Reduction of Dicer impairs Schwann cell differentiation and myelination.. J. Neurosci. Res..

[63] Ehrmann A.S., Zadro A., Tausch E., Schneider C., Stilgenbauer S., Mertens D. (2024). The NOTCH1 and miR-34a signaling network is affected by *TP53* alterations in CLL.. Leuk. Lymphoma.

[64] Ghafouri-Fard S., Bahroudi Z., Shoorei H., Abak A., Ahin M., Taheri M. (2021). microRNA-140: A miRNA with diverse roles in human diseases.. Biomed. Pharmacother..

[65] Barutta F., Bellini S., Guarrera S., Matullo G., Schalkwijk C., Stehouwer C.D., Chaturvedi N., Soedamah-Muthu S.S., Durazzo M., Gruden G. (2022). Association of serum MicroRNA-145-5p levels with microvascular complications of type 1 Diabetes: The EURODIAB prospective complications study.. Diabetes Res. Clin. Pract..

[66] Xu J., Zheng B., Ma Y., Zhang X., Cheng J., Yang J., Li P., Zhang J., Jing L., Xu F. (2023). PI3K-AKT-mTOR signaling pathway regulates autophagy of hippocampal neurons in diabetic rats with chronic unpredictable mild stress.. Behav. Brain Res..

[67] Vithian K., Hurel S. (2010). Microvascular complications: Pathophysiology and management.. Clin. Med..

[68] Østergaard L., Finnerup N.B., Terkelsen A.J., Olesen R.A., Drasbek K.R., Knudsen L., Jespersen S.N., Frystyk J., Charles M., Thomsen R.W., Christiansen J.S., Beck-Nielsen H., Jensen T.S., Andersen H. (2015). The effects of capillary dysfunction on oxygen and glucose extraction in diabetic neuropathy.. Diabetologia.

[69] Barrett E.J., Liu Z., Khamaisi M., King G.L., Klein R., Klein B.E.K., Hughes T.M., Craft S., Freedman B.I., Bowden D.W., Vinik A.I., Casellini C.M. (2017). Diabetic microvascular disease: An endocrine society scientific statement.. J. Clin. Endocrinol. Metab..

[70] Kaur P., Kotru S., Singh S., Munshi A. (2022). Role of miRNAs in diabetic neuropathy: Mechanisms and possible interventions.. Mol. Neurobiol..

[71] Le C.T., Le T.N-Y., Nguyen T.A. (2023). The pre-miRNA cleavage assays for DICER.. Methods in Enzymology.

[72] Bhattacharya S., Aggarwal R., Pal Singh V., Ramachandran S., Datta M. (2015). Downregulation of miRNAs during delayed wound healing in diabetes: Role of dicer.. Mol. Med..

[73] Zheng Y.L., Su X., Chen Y.M., Guo J.B., Song G., Yang Z., Chen P.J., Wang X.Q. (2022). microRNA-based network and pathway analysis for neuropathic pain in rodent models.. Front. Mol. Biosci..

[74] Riolo G., Cantara S., Marzocchi C., Ricci C. (2020). miRNA Targets: From prediction tools to experimental validation.. Methods Protoc..

[75] Kuhn D.E., Martin M.M., Feldman D.S., Terry A.V., Nuovo G.J., Elton T.S. (2008). Experimental validation of miRNA targets.. Methods.

[76] Friedli I., Baid-Agrawal S., Unwin R., Morell A., Johansson L., Hockings P.D. (2023). Magnetic resonance imaging in clinical trials of diabetic kidney disease.. J. Clin. Med..

[77] Koca T.T. (2015). Concomitance of diabetic neuropathic amyotrophy and cachexia: A case report with review of the literature.. North Clin Istanb.

[78] Keikha R., Hashemi-Shahri S.M., Jebali A. (2021). The relative expression of miR-31, miR-29, miR-126, and miR-17 and their mRNA targets in the serum of COVID-19 patients with different grades during hospitalization.. Eur. J. Med. Res..

[79] Wang P., Yang J., Dai S., Gao P., Qi Y., Zhao X., Liu J., Wang Y., Gao Y. (2024). miRNA-193a-mediated WT1 suppression triggers podocyte injury through activation of the EZH2/β-catenin/NLRP3 pathway in children with diabetic nephropathy.. Exp. Cell Res..

[80] Xu J., Bai S., Cao Y., Liu L., Fang Y., Du J., Luo L., Chen M., Shen B., Zhang Q. (2020). miRNA-221-3p in endothelial progenitor cell-derived exosomes accelerates skin wound healing in diabetic mice.. Diabetes Metab. Syndr. Obes..

[81] McArthur K., Feng B., Wu Y., Chen S., Chakrabarti S. (2011). MicroRNA-200b regulates vascular endothelial growth factor-mediated alterations in diabetic retinopathy.. Diabetes.

[82] Zhao X., Ling F., Zhang G., Yu N., Yang J., Xin X. (2022). The correlation between micrornas and diabetic retinopathy.. Front. Immunol..

[83] Kot A., Kaczmarek R. (2022). Exosomal miRNA profiling in vitreous humor in proliferative diabetic retinopathy.. Cells.

[84] Barsanti C., Lenzarini F., Kusmic C. (2015). Diagnostic and prognostic utility of non-invasive imaging in diabetes management.. World J. Diabetes.

[85] Dhas Y., Arshad N., Biswas N., Jones L., Ashili S. (2023). MicroRNA-21 silencing in diabetic nephropathy: Insights on therapeutic strategies.. Biomedicines.

[86] Latini A., Benedittis G.D., Ciccacci C., Novelli G., Spallone V., Borgiani P. (2024). Low expression levels of miRNA-155 and miRNA-499a are associated with obesity in Type 2 diabetes.. Epigenomics.

[87] Wang L., Wang B., Geng X., Guo X., Wang T., Xu J., Jiang L., Zhen H. (2024). microRNA-125b-5p alleviated CCI-induced neuropathic pain and modulated neuroinflammation *via* targeting SOX11.. Synapse.

[88] Jankauskas S.S., Gambardella J., Sardu C., Lombardi A., Santulli G. (2021). Functional role of miR-155 in the pathogenesis of diabetes mellitus and its complications.. Noncoding RNA.

[89] Liu Y., Gao G., Yang C., Zhou K., Shen B., Liang H., Jiang X. (2014). Stability of miR-126 in urine and its potential as a biomarker for renal endothelial injury with diabetic nephropathy.. Int. J. Endocrinol..

[90] Li Y., Tan P., Liu Q., Liu M., Wang Y., Kong W., Sun H., Shao X. (2024). MiRNA-133a-3p attenuates renal tubular epithelial cell injury *via* targeting malm1 and suppressing the notch signaling pathway in diabetic nephropathy.. Cell Biochem. Biophys..

[91] Cao Y., Cao X., Sun L., Li Y. (2019). miR-206 inhibits cell proliferation and extracellular matrix accumulation by targeting hypoxia-inducible factor 1-alpha (hif-1α) in mesangial cells treated with high glucose.. Med. Sci. Monit..

[92] Chien H.Y., Lee T.P., Chen C.Y., Chiu Y.H., Lin Y.C., Lee L.S., Li W.C. (2015). Circulating microRNA as a diagnostic marker in populations with type 2 diabetes mellitus and diabetic complications.. J. Chin. Med. Assoc..

[93] Chao Y., Gu T., Zhang Z., Wu T., Wang J., Bi Y. (2023). The role of miRNAs carried by extracellular vesicles in type 2 diabetes and its complications.. J. Diabetes.

[94] He J., Ren W., Wang W., Han W., Jiang L., Zhang D., Guo M. (2022). Exosomal targeting and its potential clinical application.. Drug Deliv. Transl. Res..

[95] Lu X., Xu R., Dong X., Bai D., Ji W., Chen X., Chen H., Hou C., Gao J. (2025). Cell-derived exosome therapy for diabetic peripheral neuropathy: A preclinical animal studies systematic review and meta-analysis.. Stem Cell Res. Ther..

[96] Heo J.I., Ryu J. (2024). Exosomal noncoding RNA: A potential therapy for retinal vascular diseases.. Mol. Ther. Nucleic Acids.

[97] Akram R., Anwar H., Javed M.S., Rasul A., Imran A., Malik S.A., Raza C., Khan I.U., Sajid F., Iman T., Sun T., Han H.S., Hussain G. (2022). Axonal regeneration: Underlying molecular mechanisms and potential therapeutic targets.. Biomedicines.

[98] Wang L., Lu X., Szalad A., Liu X.S., Zhang Y., Wang X., Golembieski W.A., Powell B., Mccann M., Lu M., Chopp M., Zhang Z.G. (2024). Schwann cell-derived exosomes ameliorate peripheral neuropathy induced by ablation of dicer in Schwann cells.. Front. Cell. Neurosci..

[99] Jia L., Chopp M., Wang L., Lu X., Szalad A., Zhang Z.G. (2018). Exosomes derived from high-glucose-stimulated Schwann cells promote development of diabetic peripheral neuropathy.. FASEB J..

[100] Ghosh M., Pearse D.D. (2023). Schwann cell-derived exosomal vesicles: A promising therapy for the injured spinal cord.. Int. J. Mol. Sci..

[101] Zhang X., Zhang J., Ren Y., Sun R., Zhai X. (2024). Unveiling the pathogenesis and therapeutic approaches for diabetic nephropathy: Insights from panvascular diseases.. Front. Endocrinol..

[102] Singh Grewal A., Bhardwaj S., Pandita D., Lather V., Singh Sekhon B. (2015). Updates on aldose reductase inhibitors for management of diabetic complications and non-diabetic diseases.. Mini Rev. Med. Chem..

[103] Chong Z.Z., Souayah N. (2025). Crumbling pathogenesis and biomarkers for diabetic peripheral neuropathy.. Biomedicines.

[104] Kato M., Natarajan R. (2015). MicroRNAs in diabetic nephropathy: Functions, biomarkers, and therapeutic targets.. Ann. N. Y. Acad. Sci..

[105] Rezaee A., Rahmanian P., Nemati A., Sohrabifard F., Karimi F., Elahinia A., Ranjbarpazuki A., Lashkarbolouki R., Dezfulian S., Zandieh M.A., Salimimoghadam S., Nabavi N., Rashidi M., Taheriazam A., Hashemi M., Hushmandi K. (2024). NF-ĸB axis in diabetic neuropathy, cardiomyopathy and nephropathy: A roadmap from molecular intervention to therapeutic strategies.. Heliyon.

[106] Kato M., Natarajan R. (2012). MicroRNA circuits in transforming growth factor-β actions and diabetic nephropathy.. Semin. Nephrol..

[107] Burk U., Schubert J., Wellner U., Schmalhofer O., Vincan E., Spaderna S., Brabletz T. (2008). A reciprocal repression between ZEB1 and members of the miR-200 family promotes EMT and invasion in cancer cells.. EMBO Rep..

[108] Tu C., Wei L., Wang L., Tang Y. (2022). Eight differential mirnas in dn identified by microarray analysis as novel biomarkers.. Diabetes Metab. Syndr. Obes..

[109] Thomas H.Y., Ford Versypt A.N. (2022). Pathophysiology of mesangial expansion in diabetic nephropathy: Mesangial structure, glomerular biomechanics, and biochemical signaling and regulation.. J. Biol. Eng..

[110] Aghaei-Zarch S.M. (2024). Crosstalk between MiRNAs/lncRNAs and PI3K/AKT signaling pathway in diabetes mellitus: Mechanistic and therapeutic perspectives.. Noncoding RNA Res..

[111] Chawra H.S., Agarwal M., Mishra A., Chandel S.S., Singh R.P., Dubey G., Kukreti N., Singh M. (2024). MicroRNA-21's role in PTEN suppression and PI3K/AKT activation: Implications for cancer biology.. Pathol. Res. Pract..

[112] Bera A., Das F., Ghosh-Choudhury N., Mariappan M.M., Kasinath B.S., Ghosh Choudhury G. (2017). Reciprocal regulation of miR-214 and PTEN by high glucose regulates renal glomerular mesangial and proximal tubular epithelial cell hypertrophy and matrix expansion.. Am. J. Physiol. Cell Physiol..

[113] Pham V.M. (2024). Targeting PI3K/AKT and MEK/ERK pathways for synergic effects on improving features of peripheral diabetic neuropathy.. J. Diabetes Investig..

[114] Liu Y., Ao X., Ding W., Ponnusamy M., Wu W., Hao X., Yu W., Wang Y., Li P., Wang J. (2018). Critical role of FOXO3a in carcinogenesis.. Mol. Cancer.

[115] Nahum-Ankonina O., Kurtzwald-Josefson E., Ciechanover A., Waldman M., Shwartz-Rohaker O., Hochhauser E., Meyer S.J., Aravot D., Phillip M., Barac Y.D. (2023). Ubiquitin proteasome system role in diabetes-induced cardiomyopathy.. Int. J. Mol. Sci..

[116] Fhu C.W., Ali A. (2021). Dysregulation of the ubiquitin proteasome system in human malignancies: A window for therapeutic intervention.. Cancers.

[117] Kim Y., Kim E.K., Chey Y., Song M.J., Jang H.H. (2023). Targeted protein degradation: Principles and applications of the proteasome.. Cells.

[118] Putta S., Lanting L., Sun G., Lawson G., Kato M., Natarajan R. (2012). Inhibiting microRNA-192 ameliorates renal fibrosis in diabetic nephropathy.. J. Am. Soc. Nephrol..

[119] Xu H., Sun F., Li X., Sun L. (2018). Down-regulation of miR-23a inhibits high glucose-induced EMT and renal fibrogenesis by up-regulation of SnoN.. Hum. Cell.

[120] Zhang H., Luo W., Sun Y., Qiao Y., Zhang L., Zhao Z., Lv S. (2016). Wnt/β-Catenin signaling mediated-uch-l1 expression in podocytes of diabetic nephropathy.. Int. J. Mol. Sci..

[121] Sulaiman M.K. (2019). Diabetic nephropathy: Recent advances in pathophysiology and challenges in dietary management.. Diabetol. Metab. Syndr..

[122] Fiorentino L., Cavalera M., Mavilio M., Conserva F., Menghini R., Gesualdo L., Federici M. (2013). Regulation of TIMP3 in diabetic nephropathy: A role for microRNAs.. Acta Diabetol..

[123] Casagrande V., Federici M., Menghini R. (2021). TIMP3 involvement and potentiality in the diagnosis, prognosis and treatment of diabetic nephropathy.. Acta Diabetol..

[124] Mahl C., Egea V., Megens R.T.A., Pitsch T., Santovito D., Weber C., Ries C. (2016). RECK (reversion-inducing cysteine-rich protein with Kazal motifs) regulates migration, differentiation and Wnt/β-catenin signaling in human mesenchymal stem cells.. Cell. Mol. Life Sci..

[125] Feng Y., Chen L., Luo Q., Wu M., Chen Y., Shi X. (2018). Involvement of microRNA-146a in diabetic peripheral neuropathy through the regulation of inflammation.. Drug Des. Devel. Ther..

[126] Qadir M.M.F., Klein D., Álvarez-Cubela S., Domínguez-Bendala J., Pastori R.L. (2019). The role of micrornas in diabetes-related oxidative stress.. Int. J. Mol. Sci..

[127] Qin W., Chung A.C.K., Huang X.R., Meng X.M., Hui D.S.C., Yu C.M., Sung J.J.Y., Lan H.Y. (2011). TGF-β/Smad3 signaling promotes renal fibrosis by inhibiting miR-29.. J. Am. Soc. Nephrol..

[128] Lin C.J., Lan Y.M., Ou M.Q., Ji L.Q., Lin S.D. (2019). Expression of miR-217 and HIF-1α/VEGF pathway in patients with diabetic foot ulcer and its effect on angiogenesis of diabetic foot ulcer rats.. J. Endocrinol. Invest..

[129] Yu C.Y., Yang C.Y., Rui Z.L. (2019). MicroRNA-125b-5p improves pancreatic β-cell function through inhibiting JNK signaling pathway by targeting DACT1 in mice with type 2 diabetes mellitus.. Life Sci..

[130] Nigi L., Grieco G.E., Ventriglia G., Brusco N., Mancarella F., Formichi C., Dotta F., Sebastiani G. (2018). MicroRNAs as regulators of insulin signaling: Research updates and potential therapeutic perspectives in type 2 diabetes.. Int. J. Mol. Sci..

[131] Jurj A., Fontana B., Varani G., Calin G.A. (2024). Small molecules targeting microRNAs: New opportunities and challenges in precision cancer therapy.. Trends Cancer.

[132] Liu F., Lou Y.L., Wu J., Ruan Q.F., Xie A., Guo F., Cui S.P., Deng Z.F., Wang Y. (2012). Upregulation of microRNA-210 regulates renal angiogenesis mediated by activation of VEGF signaling pathway under ischemia/perfusion injury *in vivo* and *in vitro* .. Kidney Blood Press. Res..

[133] Wen W., Wei Y., Gao S. (2023). Functional nucleic acids for the treatment of diabetic complications.. Nanoscale Adv..

[134] Bijkerk R., de Bruin R.G., van Solingen C., van Gils J.M., Duijs J.M.G.J., van der Veer E.P., Rabelink T.J., Humphreys B.D., van Zonneveld A.J. (2016). Silencing of microRNA-132 reduces renal fibrosis by selectively inhibiting myofibroblast proliferation.. Kidney Int..

[135] Liczner C., Duke K., Juneau G., Egli M., Wilds C.J. (2021). Beyond ribose and phosphate: Selected nucleic acid modifications for structure–function investigations and therapeutic applications.. Beilstein J. Org. Chem..

[136] Negeem Z., Abdel Moneim A., Mahmoud B., Ahmed A.E., Abd El-Hameed A.M., Eskandrani A.A., Hasona N.A. (2024). The implication of miR-200a and miR-132 expression and their correlations with NF-κB/TNF-alpha signaling in adults with diabetic nephropathy.. Saudi J. Biol. Sci..

[137] Wang B., Yao K., Wise A.F., Lau R., Shen H.H., Tesch G.H., Ricardo S.D. (2017). *miR-378* reduces mesangial hypertrophy and kidney tubular fibrosis *via* MAPK signalling.. Clin. Sci..

[138] Koga K., Yokoi H., Mori K., Kasahara M., Kuwabara T., Imamaki H., Ishii A., Mori K.P., Kato Y., Ohno S., Toda N., Saleem M.A., Sugawara A., Nakao K., Yanagita M., Mukoyama M. (2015). MicroRNA-26a inhibits TGF-β-induced extracellular matrix protein expression in podocytes by targeting CTGF and is downregulated in diabetic nephropathy.. Diabetologia.

[139] Zhang Y., Sun X., Icli B., Feinberg M.W. (2017). Emerging roles for micrornas in diabetic microvascular disease: Novel targets for therapy.. Endocr. Rev..

[140] Smyth A., Callaghan B., Willoughby C.E., O’Brien C. (2022). The role of mir-29 family in tgf-β driven fibrosis in glaucomatous optic neuropathy.. Int. J. Mol. Sci..

[141] Shi J., Liu X., Jiao Y., Tian J., An J., Zou G., Zhuo L. (2025). MTOR pathway: A key player in diabetic nephropathy progression and therapeutic targets.. Genes Dis..

[142] Liu X., Zhang Y., Liang H., Zhang Y., Xu Y. (2020). microRNA-499-3p inhibits proliferation and promotes apoptosis of retinal cells in diabetic retinopathy through activation of the TLR4 signaling pathway by targeting IFNA2.. Gene.

[143] Gao C., Yang T., Shu J., Gao X., Meng C. (2025). Overexpression of miR-133a-3p reduces microglia activation by binding to GCH1, alleviating neuroinflammation and neuropathic pain.. Exp. Brain Res..

[144] Sanguineti R., Puddu A., Nicolò M., Traverso C.E., Cordera R., Viviani G.L., Maggi D. (2021). MIR-126 MIMIC counteracts the increased secretion of vegf-a induced by high glucose in ARPE-19 cells.. J. Diabetes Res..

[145] Wu Y., Xu D., Zhu X., Yang G., Ren M. (2017). MiR-106a associated with diabetic peripheral neuropathy through the regulation of 12/15-LOX-mediated oxidative/nitrative stress.. Curr Neurovasc Res.

[146] El-Lithy G.M., El-Bakly W.M., Matboli M., Abd-Alkhalek H.A., Masoud S.I., Hamza M. (2016). Prophylactic L-arginine and ibuprofen delay the development of tactile allodynia and suppress spinal miR-155 in a rat model of diabetic neuropathy.. Transl. Res..

[147] Conte G., Nguyen N.T., Alves M., de Diego-Garcia L., Kenny A., Nicke A., Henshall D.C., Jimenez-Mateos E.M., Engel T. (2020). P2X7 Receptor-Dependent microrna expression profile in the brain following status epilepticus in mice.. Front. Mol. Neurosci..

[148] Chang L.L., Wang H.C., Tseng K.Y., Su M.P., Wang J.Y., Chuang Y.T., Wang Y.H., Cheng K.I. (2020). Upregulation of miR-133a-3p in the sciatic nerve contributes to neuropathic pain development.. Mol. Neurobiol..

[149] Wu X., Wang X., Yin Y., Zhu L., Zhang F., Yang J. (2021). Investigation of the role of miR-221 in diabetic peripheral neuropathy and related molecular mechanisms.. Adv. Clin. Exp. Med..

[150] Hashemi M., Zandieh M.A., Ziaolhagh S., Mojtabavi S., Sadi F.H., Koohpar Z.K., Ghanbarirad M., Haghighatfard A., Behroozaghdam M., Khorrami R., Nabavi N., Ren J., Reiter R.J., Salimimoghadam S., Rashidi M., Hushmandi K., Taheriazam A., Entezari M. (2023). Nrf2 signaling in diabetic nephropathy, cardiomyopathy and neuropathy: Therapeutic targeting, challenges and future prospective.. Biochim. Biophys. Acta Mol. Basis Dis..

[151] Wang Z., Liu F., Wei M., Qiu Y., Ma C., Shen L., Huang Y. (2018). Chronic constriction injury-induced microRNA-146a-5p alleviates neuropathic pain through suppression of IRAK1/TRAF6 signaling pathway.. J. Neuroinflammation.

[152] Pang B., Qiao L., Wang S., Guo X., Xie Y., Han L. (2021). MiR-214-3p plays a protective role in diabetic neuropathic rats by regulating Nav1.3 and TLR4.. Cell Biol. Int..

[153] Wu Y., Gu Y., Shi B. (2020). miR-590-3p Alleviates diabetic peripheral neuropathic pain by targeting RAP1A and suppressing infiltration by the T cells.. Acta Biochim. Pol..

[154] Reid K.M., Sanchez-Nieto J.M., Terrasse S., Faccenda D., Pernaute B., Campanella M., Rodriguez T.A., Cobb B.S. (2023). MicroRNAs Regulate Ca^2+^ homeostasis in murine embryonic stem cells.. Cells.

[155] Motti D., Lerch J.K., Danzi M.C., Gans J.H., Kuo F., Slepak T.I., Bixby J.L., Lemmon V.P. (2017). Identification of mi RNA s involved in DRG neurite outgrowth and their putative targets.. FEBS Lett..

[156] Sun W., Zhang L., Li R. (2017). Overexpression of miR-206 ameliorates chronic constriction injury-induced neuropathic pain in rats *via* the MEK/ERK pathway by targeting brain-derived neurotrophic factor.. Neurosci. Lett..

[157] Zhang P., Wang C., Li C., Wang J. (2024). miR-34a-5p predicts the risk of diabetic neuropathic pain and mediates neuroinflammation in microglia *via* targeting ENPP3.. Immunol. Invest..

[158] Kong J., Liu L., Song L., Zhao R., Feng Y. (2023). MicroRNA miR-34a-5p inhibition restrains oxidative stress injury of macrophages by targeting MDM4.. Vascular.

[159] Li M., Li H., Liu X., Xu D., Wang F. (2016). MicroRNA-29b regulates TGF-β1-mediated epithelial–mesenchymal transition of retinal pigment epithelial cells by targeting AKT2.. Exp. Cell Res..

[160] Ciccacci C., Morganti R., Di Fusco D., D’Amato C., Cacciotti L., Greco C., Rufini S., Novelli G., Sangiuolo F., Marfia G.A., Borgiani P., Spallone V. (2014). Common polymorphisms in MIR146a, MIR128a and MIR27a genes contribute to neuropathy susceptibility in type 2 diabetes.. Acta Diabetol..

[161] Piona C., Costantini S., Zusi C., Cozzini T., Pedrotti E., Marigliano M., Fornari E., Maguolo A., Morandi A., Maffeis C. (2022). Early marker of ocular neurodegeneration in children and adolescents with type 1 diabetes: The contributing role of polymorphisms in mir146a and mir128a genes.. Acta Diabetol..

[162] Chang W.W., Wen L.Y., Zhang L., Tong X., Jin Y.L., Chen G.M. (2022). Association of rs2910164 in miR-146a with type 2 diabetes mellitus: A case–control and meta-analysis study.. Front. Endocrinol..

[163] Li Y., Ma W., Xie C., Zhang M., Yin X., Wang F., Xu J., Shi B. (2016). Identification of genes and signaling pathways associated with diabetic neuropathy using a weighted correlation network analysis.. Medicine.

[164] Liu J.C., Xue D.F., Wang X.Q., Ai D.B., Qin P.J. (2019). MiR-101 relates to chronic peripheral neuropathic pain through targeting KPNB1 and regulating NF-κB signaling.. Kaohsiung J. Med. Sci..

[165] Ashjari D., Karamali N., Rajabinejad M., Hassani S.S., Afshar Hezarkhani L., Afshari D., Gorgin Karaji A., Salari F., Rezaiemanesh A. (2022). The axis of long non-coding RNA MALAT1/miR-1-3p/CXCR4 is dysregulated in patients with diabetic neuropathy.. Heliyon.

[166] Leinders M., Üçeyler N., Pritchard R.A., Sommer C., Sorkin L.S. (2016). Increased miR-132-3p expression is associated with chronic neuropathic pain.. Exp. Neurol..

[167] Heyn J., Luchting B., Hinske L.C., Hübner M., Azad S.C., Kreth S. (2016). miR-124a and miR-155 enhance differentiation of regulatory T cells in patients with neuropathic pain.. J. Neuroinflammation.

[168] Fang Y., Sun S., Wu J., Liu G., Wu Q., Ran X. (2024). Alterations in the levels of urinary exosomal microrna-183-5p and microrna-125a-5p in individuals with type 2 diabetes mellitus.. Biomedicines.

[169] Saha P., Yarra S.S., Arruri V., Mohan U., Kumar A. (2024). Exploring the role of mirna in diabetic neuropathy: From diagnostics to therapeutics.. Naunyn Schmiedebergs Arch. Pharmacol..

[170] Xourgia E., Papazafiropoulou A., Melidonis A. (2018). Circulating microRNAs as biomarkers for diabetic neuropathy: A novel approach.. World J. Exp. Med..

[171] Szydełko J., Czop M., Petniak A., Lenart-Lipińska M., Kocki J., Zapolski T., Matyjaszek-Matuszek B. (2024). Identification of plasma miR-4505, miR-4743-5p and miR-4750-3p as novel diagnostic biomarkers for coronary artery disease in patients with type 2 diabetes mellitus: A case-control study.. Cardiovasc. Diabetol..

[172] Momin M.Y., Gaddam R.R., Kravitz M., Gupta A., Vikram A. (2021). The challenges and opportunities in the development of microrna therapeutics: A multidisciplinary viewpoint.. Cells.

[173] Andersen H.H., Duroux M., Gazerani P. (2014). MicroRNAs as modulators and biomarkers of inflammatory and neuropathic pain conditions.. Neurobiol. Dis..

[174] Banack S.A., Dunlop R.A., Mehta P., Mitsumoto H., Wood S.P., Han M., Cox P.A. (2024). A microRNA diagnostic biomarker for amyotrophic lateral sclerosis.. Brain Commun..

[175] Li X., Dai A., Tran R., Wang J. (2023). Text mining-based identification of promising miRNA biomarkers for diabetes mellitus.. Front. Endocrinol..

[176] Aghamiri S., Talaei S., Ghavidel A.A., Zandsalimi F., Masoumi S., Hafshejani N.H., Jajarmi V. (2020). Nanoparticles-mediated CRISPR/Cas9 delivery: Recent advances in cancer treatment.. J. Drug Deliv. Sci. Technol..

[177] Condrat C.E., Thompson D.C., Barbu M.G., Bugnar O.L., Boboc A., Cretoiu D., Suciu N., Cretoiu S.M., Voinea S.C. (2020). Mirnas as biomarkers in disease: Latest findings regarding their role in diagnosis and prognosis.. Cells.

[178] Ijee S., Chambayil K., Chaudhury A.D., Bagchi A., Modak K., Das S., Benjamin E.S.B., Rani S., Paul D.Z., Nath A., Roy D., Palani D., Priyanka S., Ravichandran R., Kumary B.K., Sivamani Y., S V., Babu D., Nakamura Y., Thamodaran V., Balasubramanian P., Velayudhan S.R. (2024). Efficient deletion of microRNAs using CRISPR/Cas9 with dual guide RNAs.. Front. Mol. Biosci..

[179] Chen Q., Chen J., Liu Y.N., Qi S.H., Huang L.Y. (2023). Exosome-based drug delivery systems for the treatment of diabetes and its complications: Current opinion.. Extracellular Vesicles and Circulating Nucleic Acids.

[180] Lee S.W.L., Paoletti C., Campisi M., Osaki T., Adriani G., Kamm R.D., Mattu C., Chiono V. (2019). MicroRNA delivery through nanoparticles.. J. Control. Release.

[181] Lam J.K.W., Chow M.Y.T., Zhang Y., Leung S.W.S. (2015). Sirna versus mirna as therapeutics for gene silencing.. Mol. Ther. Nucleic Acids.

[182] Chehelgerdi M., Chehelgerdi M., Allela O.Q.B., Pecho R.D.C., Jayasankar N., Rao D.P., Thamaraikani T., Vasanthan M., Viktor P., Lakshmaiya N., Saadh M.J., Amajd A., Abo-Zaid M.A., Castillo-Acobo R.Y., Ismail A.H., Amin A.H., Akhavan-Sigari R. (2023). Progressing nanotechnology to improve targeted cancer treatment: Overcoming hurdles in its clinical implementation.. Mol. Cancer.

[183] Tajabadi Z., Dadkhah P., Gholami Chahkand M.S., Esmaeilpour Moallem F., Karimi M.A., Amini-Salehi E., Karimi M. (2025). Exploring the role of exosomes in diabetic neuropathy: From molecular mechanisms to therapeutic potential.. Biomed. Pharmacother..

